# Burden of dengue, leishmaniasis and lymphatic filariasis in India and its states from 1990–2019: Analysis from the Global Burden of Disease study (GBD 2019)

**DOI:** 10.1371/journal.pone.0292723

**Published:** 2023-10-18

**Authors:** Omprokash Dutta, Ajay Prasanth, Ashu Kumari, Kumari Akanksha, Farah Deeba, Nasir Salam

**Affiliations:** 1 Department of Microbiology, Central University of Punjab, Bathinda, India; 2 Centre for Interdisciplinary Research in Basic Sciences, Jamia Millia Islamia, New Delhi, India; 3 Department of Biosciences, Jamia Millia Islamia, New Delhi, India; Berhampur University, INDIA

## Abstract

Vector-borne diseases such as dengue, leishmaniasis, and lymphatic filariasis, constitute significant sources of illness, disability, and mortality among the poor and vulnerable in many countries around the world, including India. Based on the global burden of diseases, injuries, and risk factors study 2019, we analyse the burden of dengue, leishmaniasis, and lymphatic filariasis, in India from 1990 to 2019. Over this period, there was a reduction in the burden of lymphatic filariasis and leishmaniasis. Notably, dengue emerged as the most common vector-borne disease, exhibiting high fatality rate above 15 years of age and the highest DALY within 15–49 age group. Additionally, dengue cases surged substantially between 1990 and 2019. Leishmaniasis related mortality and DALY declined in the year 2019 compared to the year 1990, with high mortality and DALY in the 0-49-year-old age group. For lymphatic filariasis, DALY was more pronounce among those in the 15–49-year age group, which underwent reduction in 2019. Males had a higher burden in other vector-borne diseases than females, although females had a slightly elevated dengue burden. These findings highlight the evolving epidemiological trends related to vector-borne diseases in India, over the last three decades and underline the critical significance of sustained efforts for the elimination and control of vector-borne diseases.

## Introduction

Emerging and re-emerging illnesses spread by arthropod vectors represent a major public health concern around the world [1]. Infections transmitted by mosquitoes, sandflies, and other insects are collectively referred to as vector-borne diseases (VBDs). They account for 17% of the globally estimated burden of all infectious diseases, and approximately 82% of the world’s population lives in areas where they are at risk from at least one VBD [2, 3]. More than a billion cases and one million deaths globally are attributed to VBDs. Furthermore, they are also responsible for one-sixth of global disability and diseases each year [4, 5]. Many of these VBDs, such as dengue, leishmaniasis, and lymphatic filariasis (LF) are also categorized as Neglected Tropical Diseases (NTDs) and they represent a significant portion of disability-adjusted life years (DALYs) attributed to infectious diseases, following only AIDS [2].

Incidence and outbreaks of VBDs are frequently reported from India, due to its tropical climate and geographical distribution of vector populations [6]. India bears one of the highest absolute burdens of infectious diseases globally, with VBDs being one of the most prevalent. Within India, six different forms of VBDs are prevalent with dengue, leishmaniasis and LF being the most common apart from malaria [4].

Dengue infects an estimated 50 million individuals annually across 100 countries, with India alone accounting for 34% of the global burden [7]. In tropical and sub-tropical regions worldwide, LF affects 120 million people and India contributes to 41% of global LF cases [8]. Every year, there are 1.5 to 2 million new cases of leishmaniasis reported globally, with India accounting for 18% of this burden [9]. India launched Global Programme to Eliminate Lymphatic Filariasis (GPELF) in the year 2000 and a program to eliminate visceral leishmaniasis (VL) in the year 2005. Despite notable progress in reducing the burden of LF and leishmaniasis, recent years have witnessed an upsurge in VBDs, especially dengue, which has evolved into a severe public health concern in India.

The control and elimination of VBDs require a concerted effort involving maximising public health initiatives, resource allocation, awareness campaigns, and targeted intervention for the affected population. To achieve these goals, it is essential to analyse the present burden and evolving trends of VBDs. Such analyses serve to gauge the effectiveness of current strategies and highlight the knowledge gap required for future efforts. In this study, we utilised data from the global burden of disease 2019 (GBD) report to assess the incidence, prevalence, mortality, and DALYs associated with dengue, leishmaniasis, and LF. This assessment was conducted across different age groups, genders, and geographical regions. The aim was to understand epidemiological trends and monitor the progress of VBD control programs.

## Methods

We accessed data on VBDs from the GBD data repository. We used the information to analyse the incidence, prevalence, death, and DALYs. Our analysis focused on the temporal trends of VBDs (dengue, leishmaniasis, and LF) in India, utilising data from 1990 and 2019. The GBD India comparison tool (https://vizhub.healthdata.org/gbd-compare/india) was used to extract state-level data on the burden of VBDs.

We comprehensively analysed the extracted data to depict the burden of disease categorised by age, gender, and time, across all Indian states and union territories. We evaluated and compared prevalence, incidence, DALYs, and mortality per 100,000 population for both genders. The age-wise distribution of VBDs was determined for age groups below 5 years, 5–14 years, 15–49 years, 50–69 years, and above 70 considering data from both 1990 and 2019. Values for mortality, incidence, prevalence, and DALYs were presented as mean estimates with 95% uncertainty intervals. The data were analysed and maps were created using QGIS 3.32 software.

## Results

### Dengue

**Fig 1:**
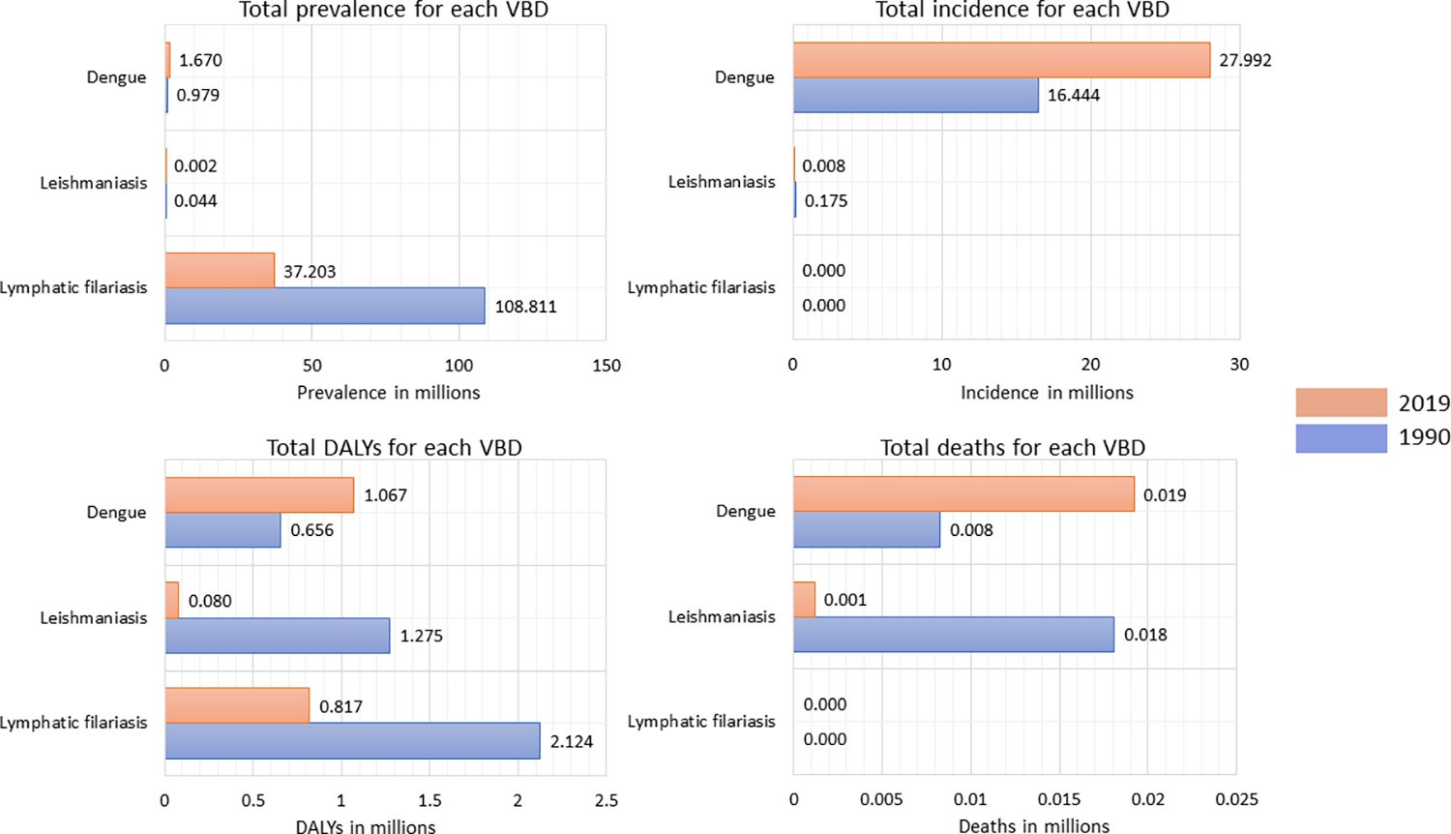
Change in total prevalence, incidence, DALYs, and death in India for individual VBDs between 1990 and 2019.

**Fig 2:**
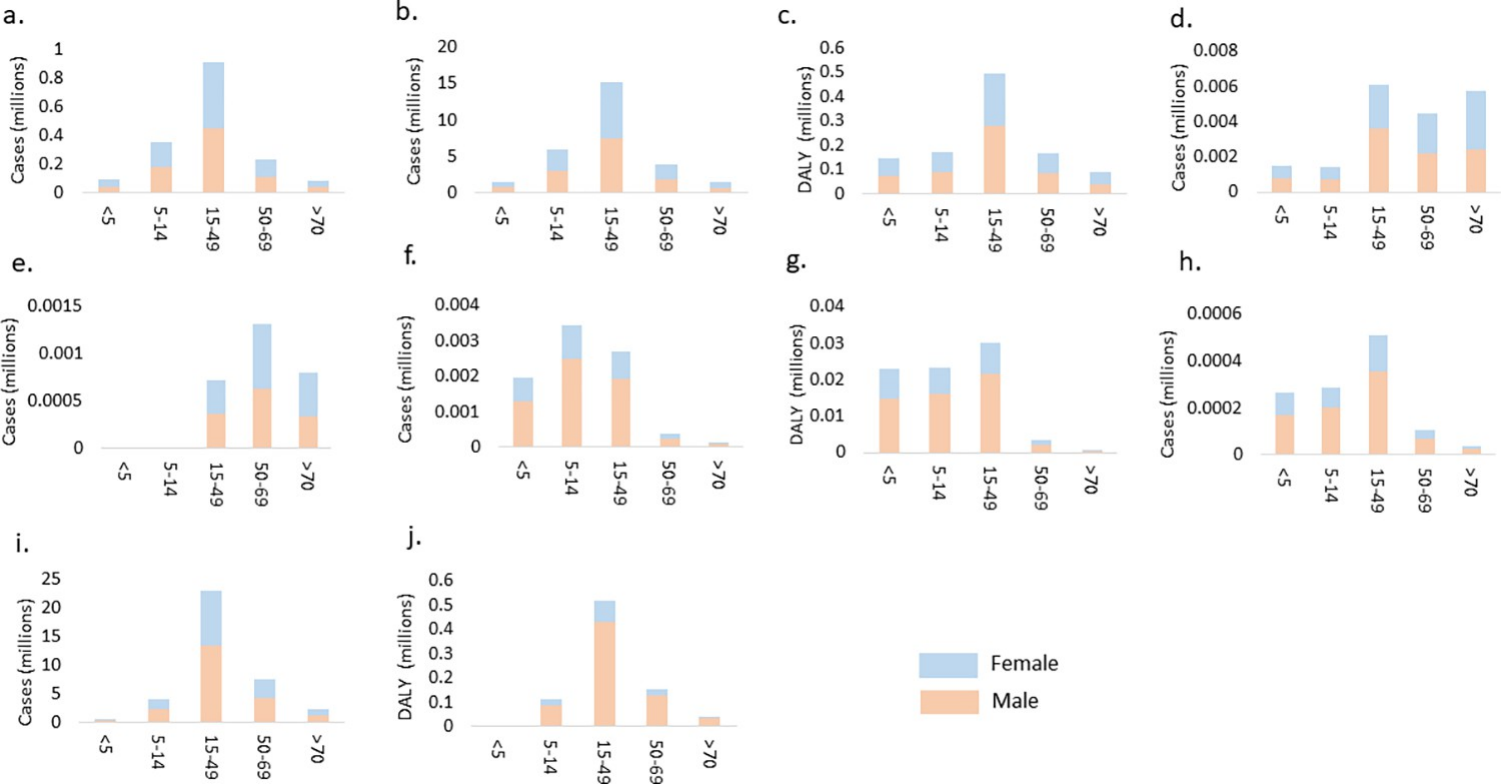
Difference by gender and age in 2019 for; Dengue a. Prevalence b. Incidence c. DALYs d. Death. Leishmaniasis e. Prevalence f. Incidence g. DALYs h. Death, LF i. Prevalence j. DALYs.

**Fig 3:**
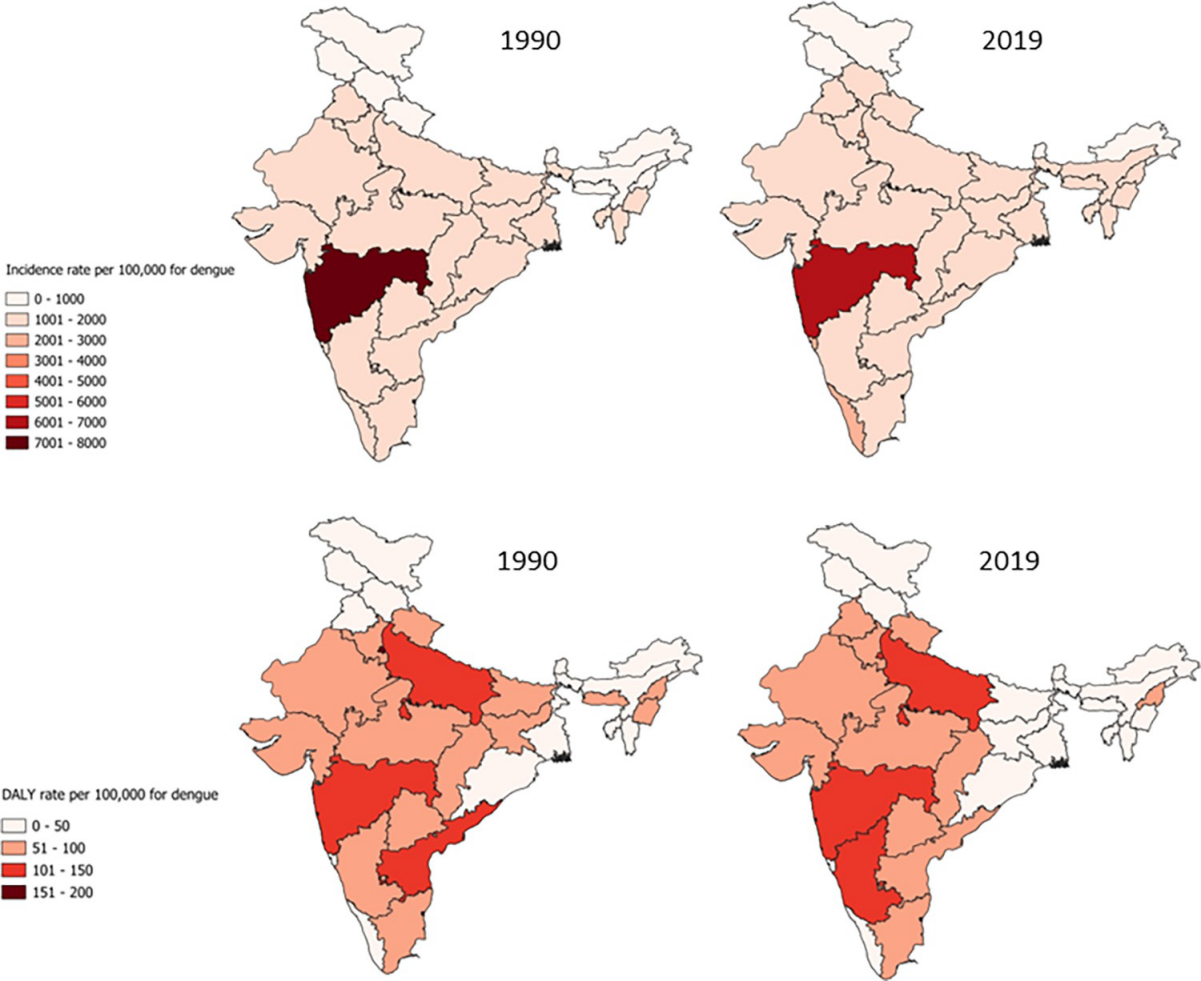
Change in rate of incidence and DALYs for dengue between 1990 and 2019.

Our analysis indicated a notable increase in incidents of dengue between 1990 to 2019, with figures rising from 16.44 million (95% UI 3.72–44.04) cases to 28.0 million (95% UI 9.43–72.6) signifying a 70% increase (Fig 1, Table 1). In terms of gender and age distribution of dengue cases, the rate of prevalence, incidence, and death were slightly higher among females as compared to males in 2019. Moreover, the 15–49 age group bore a disproportionate burden of dengue in comparison to other age groups (Fig 2, S1 Table in S1 File). Analysis of GBD data also indicated 88654 cases of dengue among children below 5 years of age. The mortality rate associated with dengue rose from 0.96 (95% UI 0.22–1.39) per 100,000 in 1990 to 1.38 (95% UI 0.26–1.81) per 100,000 in 2019 while the DALYs for dengue did not show any substantial change (S1 and S4 Figs in S1 File). There was an upward trend in the incidence of dengue with rates ascending from 1921.91 (95% UI 435.56–5147.70) cases per 100,000 in 1990 to 2012.79 (95% UI 678.32–5220.67) cases per 100,000 in 2019. The escalating trends were evident across all states except Maharashtra, Andhra Pradesh, Sikkim, and Manipur (Fig 3). The state of Maharashtra was of particular concern with around three times higher incident rate reported than Kerala, which is reporting the second highest incident rate for dengue. Interestingly, no discernible correlation emerged between the rates of prevalence, incidence, DALYs, and death attributed to dengue and the socio-demographic index (SDI) of each state (S5–S8 Figs in S1 File).

**Table 1:** Burden of dengue, leishmaniasis, and LF in India for the years 1990 and 2019. Each value represents mean estimates and data in parentheses are 95% uncertainty intervals.

Disease	**Prevalence (95% UI)**					
	Cases (millions)			Rate (per 100,000)		
	1990	2019	Percent change	1990	2019	Percent change
Dengue	0.98 (0.2–2.7)	1.67 (0.54–4.33)	70.4	114.39 (25.25–314.30)	120.12 (39.01–311.83)	5
Leishmaniasis	0.04 (0.025–0.06)	0.0028 (0.0018–0.004)	-93	4.69 (2.99–6.83)	0.2 (0.13–0.29)	-95.73
Lymphatic filariasis	108.81 (100.9–117.5)	37.20 (32.56–43.35)	-65.8	12717.75 (11800.41–13733.93)	2675.13 (2341.64–3117.78)	-79
	**Incidence (95% UI)**					
	Cases (millions)			Rate (per 100,000)		
	1990	2019	Percent change	1990	2019	Percent change
Dengue	16.44 (3.72–44.04)	28.0 (9.43–72.6)	70.31	1921.91 (435.56–5147.70)	2012.79 (678.32–5220.67)	4.73
Leishmaniasis	0.17 (0.11–0.25)	0.0086 (0.0051–0.13)	-95	20.52 (13.05–29.62)	0.62 (0.37–0.94)	-97
Lymphatic filariasis	0	0	0	0	0	0
	**DALY (95% UI)**					
	DALYs (millions)			Rate (per 100,000)		
	1990	2019	Percent change	1990	2019	Percent change
Dengue	0.65 (0.14–1.1)	1.1 (0.23–1.7)	69.23	76.71 (16.67–128.14)	76.70 (16.60–121.24)	-0.01
Leishmaniasis	1.27 (0.0034–6.65)	0.08 (0.00065–0.47)	-93.7	149.02 (0.4–777.24)	5.77 (0.05–33.8)	-96
Lymphatic filariasis	2.12 (1.41–3.02)	0.82 (0.48–1.34)	-61	248.26 (164.96–353.4)	58.71 (34.92–96.68)	-76.35
	**Deaths (95% UI)**					
	Cases (millions)			Rate (per 100,000)		
	1990	2019	Percent change	1990	2019	Percent change
Dengue	0.0083 (0.0018–0.12)	0.02 (0.0036–0.025)	141	0.96 (0.22–1.39)	1.38 (0.26–1.81)	43.75
Leishmaniasis	0.02 (0.000002–0.09)	0.0012 (0–0.0066)	-94	2.11 (0–10.57)	0.09 (0–0.48)	-95.73
Lymphatic filariasis	0	0	0	0	0	0

### Leishmaniasis

**Fig 4:**
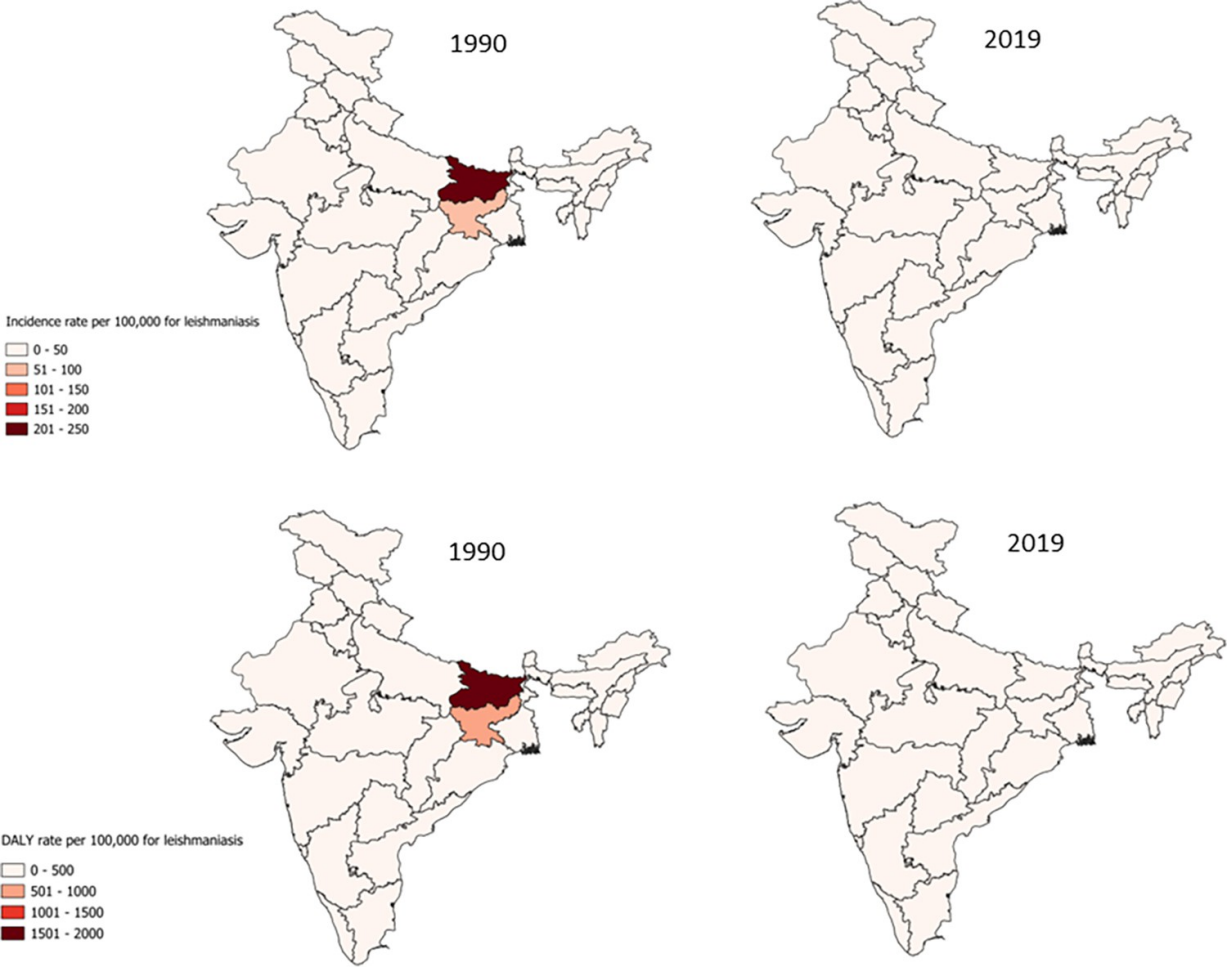
Change in rate of incidence and DALYs for leishmaniasis between 1990 and 2019.

India has made significant efforts for the elimination of leishmaniasis since 2005, with a target of reducing incidence to < 1 case per 10,000 at the subdistrict level by 2023. Despite substantial gains in the reduction of leishmaniasis incidence, that target has yet to be attained. The GBD study includes data on cases of VL, cutaneous leishmaniasis and mucocutaneous leishmaniasis. However, the study does not separately include cases of Post Kala-Azar Dermal Leishmaniasis (PKDL). Our analysis demonstrates that incidence of leishmaniasis declined by 95% from 0.17 million (95% UI 0.11–0.25) cases in 1990 to 0.0086 million (95% UI 0.0051–0.13) cases in 2019 (Fig 1). The incidence rate dropped from 20.52 (95% UI 13.05–29.62) cases per 100,000 in 1990 to 0.62 cases (95% UI 0.37–0.94) per 100,000 in 2019 (Table 1, S2 Fig in S1 File). Notably, the states of Bihar, Jharkhand, and West Bengal which bore a significant burden of leishmaniasis in India achieved a more than 95% reduction in incidence rates. However, the target of <1 case per 10,000 population remains unattained in these three states (Fig 4, S9–S12 Figs in S1 File). Analysis of 2019 prevalence data of leishmaniasis reveals that the disease impacts everyone above the age of 15 with the 50–69 years age group and females sharing the larger burden of the disease (Fig 2). Though incidence was higher in male children below the age of 15 years. There was an approximate 96% reduction in DALYs attributed to leishmaniasis from 1990 to 2019 (S2 Fig in S1 File).

### Lymphatic filariasis

**Fig 5:**
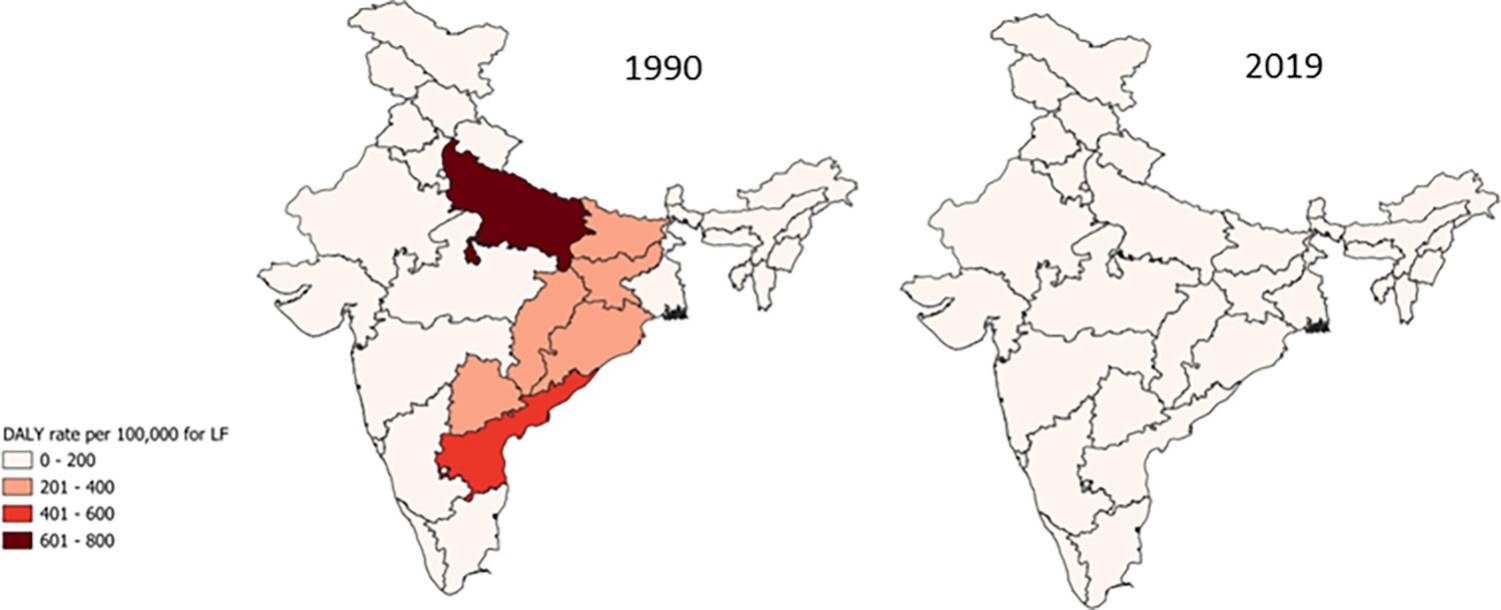
Change in rate of DALYs for LF between 1990 and 2019.

LF used to be a significant driver of permanent and long-term disability in India and made a substantial contribution significantly to the burden of neglected tropical diseases in the country. With the launch of GPELF in the year 2000 by mass drug administration, substantial progress has been made. For the years under consideration, no new cases or death were reported from LF in India. However, 37.20 (95% UI 32.56–43.35) million people were still affected by LF in 2019, contributing significantly to the overall burden of NTDs in India (Fig 1, Table 1). The state of Uttar Pradesh reported the highest prevalence rate in 2019. Overall, there was an improvement in the burden of LF with approximately a 76% decline in DALY rate per 100,000 from 248.26 (95% UI 164.96–353.4) in 1990 to 58.71 (95% UI 34.92–96.68) in 2019 (Fig 5, S3 Fig in S1 File). Males appeared to be more affected by the disease as compared to females. The 15–49 years age group was the major contributor to prevalence and 560,365 children below the age of 5 also suffered from LF (Fig 2). The states of Bihar, Jharkhand, Uttar Pradesh, Andhra Pradesh, and Odisha, with lower SDIs exhibited higher prevalence and DALYs of LF in 1990. These states also witnessed the most significant decline in both parameters by 2019 compared to states with higher SDIs (S13 and S14 Figs in S1 File).

**Fig 6:**
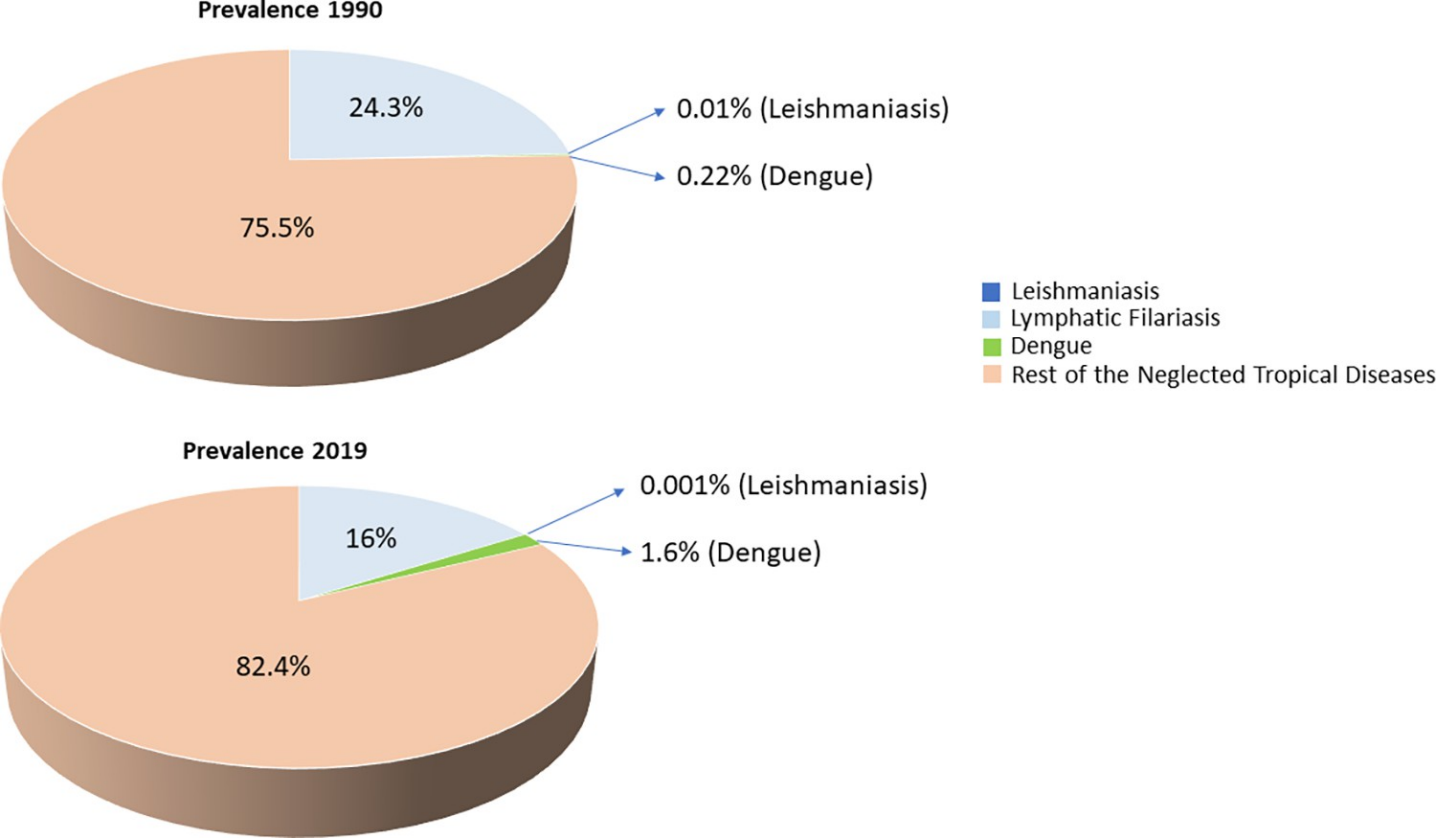
Overall contribution of dengue, leishmaniasis, and LF in total neglected tropical diseases prevalence in India for the years 1990 and 2019. Percent contribution 1990: dengue (0.22%), leishmaniasis (0.01%), lymphatic filariasis (24.3%), rest of the neglected tropical diseases (75.5%). Percent contribution 2019: dengue (1.6%), leishmaniasis (0.001%), LF (16%), rest of the neglected tropical diseases (82.4%).

Considering the overall burden of NTDs in India, LF while reduced since 1990, still contributes substantially to the burden, albeit its share has diminished. Notable, dengue has gradually emerged as a significant contributor to the overall NTD burden in India (Fig 6).

## Discussion

The current study analyses the temporal trends of three pivotal VBDs in India. While dengue has been reported for last two centuries in India, recent years have witnessed frequent outbreaks of dengue infection in both urban and rural settings [10]. All four dengue serotypes have been reported from India, alongside other vector-borne infections like chikungunya and malaria [11]. Several reports point towards underreporting of dengue cases which has potential implications for disease surveillance and preventive public health measures [12]. Our analysis highlights an increase in incidence, prevalence, death, and DALYs for dengue, paralleling the global trends of rising values in these parameters. In 2019 alone, an estimated 28 million dengue cases and nearly 20,000 related deaths were recorded, significantly higher than 157,315 cases and 166 deaths reported by the National Centre for Vector Borne Diseases Control (NCVBDC) for the same year (S2 Table in S1 File) [13]. Incidence of dengue has been often correlated with rapid urbanisation and monsoon cycles which provides an ideal breeding ground for the mosquito vector [14]. Interestingly, Maharashtra registered the highest incidence rate among all other states possibly due to urbanised infrastructure. However, DALYs and deaths were comparable to many other states indicating better management and preventive public health measures for the control of the disease. Notably, while the SDI of a state is correlated with improved health and better management, no direct correlation was observed between SDI and dengue. Low SDI states like Bihar and Uttar Pradesh were equally affected by dengue as higher SDI states such as Karnataka, Haryana, Maharashtra etc. In fact, the highest rate of death and DALY per 100,000 was observed in Delhi which is the capital with better health services and disease surveillance, underscoring the role of other contributing factors such as poorly planned infrastructure with rainfall patterns during the monsoon season. Another factor that might have resulted in observed geographic heterogeneity among different states is the variations in climactic patterns. India has six climactic zones, exhibiting variations in temperature and precipitation patterns. Earlier studies have highlighted the impact of temperature and precipitation in reducing the extrinsic incubation period of the virus as well as the fertility and longevity of the mosquito vector [15, 16]. Climate change has emerged as one of the key risk factors in the rise of the incidence of VBDs alongside urbanisation and migration of the human population. These factors will undoubtedly impact the spread and control of VBDs, particularly dengue. While the overall DALY for dengue increased by 69%, the rate per 100,000 remained consistent over a span of three decades, possibly indicating self-resolving cases without causing much disability.

India launched GPELF in 2000 and VL elimination program in 2005 to reduce the burden of LF and visceral leishmaniasis [17, 18]. Our analyses highlighted that considerable success has been achieved on both these fronts with no new cases being reported for LF and a 95% reduction in leishmaniasis incidence from 170,000 cases in 1990 to 8600 cases in 2019. However better surveillance at district level is warranted as the estimated figures remain higher than those reported by NCVBDC (S2 Table in S1 File). Despite reduced incidence, the burden of LF remains high with 38 million cases in 2019 and a cumulative DALY of 820,000. Our analyses showcases reduced prevalence in 2019 across all states and union territories as compared to 1990 with states having lower SDIs like Bihar, Jharkhand, Uttar Pradesh, Andhra Pradesh, and Odisha displaying higher prevalence as compared to states with higher SDIs, suggesting better surveillance and monitoring in these states. A recent report by Dhamnetiya et al., in 2021, which used GBD data, underlined India’s relative success in controlling leishmaniasis as compared to other endemic countries like Brazil due to better public health measures, integrated vector management, rapid diagnostic kits and safe therapeutic options like miltefosine [19]. Leishmaniasis is mostly reported in Bihar, Jharkhand, and West Bengal and all three states have reduced the burden substantially with sustained monitoring and surveillance at the district level. However, the rise of HIV-VL coinfection cases and the emergence of new endemic foci in the states of Himachal Pradesh and Kerala stress the need for sustained eradication efforts targeting leishmaniasis [20–22]. It is also important to understand the prevalence of LF-leishmaniasis coinfection due to their shared epidemiology and contrasting host immune response. Several studies have suggested a high probability of disease progression and increased severity in cases of coinfection. Mass drug administration against LF could also potentially reduce the burden of VL in endemic settings. These factors should be taken into careful consideration when designing and implementing integrated control programs for VBDs [23–25].

The present study compares and analyses the burden of the three most important VBDs in India. It underlines the progress that has been achieved in controlling endemic diseases like LF and leishmaniasis, while signalling emerging challenges in the form of new VBDs like dengue that could proves to be more fatal. VBDs are dependent on the vector population and multiple studies have pointed towards the role of climactic factors in the re-emergence of these diseases. Such environmental elements have the potential to reverse the gains made in the elimination of LF and leishmaniasis and they should be carefully considered when designing strategies for the control of VBDs. Furthermore, many public health-related programs have been interrupted due to the COVID-19 pandemic, evident in increased outbreaks of measles and polio in different parts of the world [26, 27]. Its effect on the elimination program for LF and leishmaniasis should also be analysed.

The present study is based on temporal, regional, gendered, and age-based analysis of the 2019 GBD data from India. However, it has some limitations. The accuracy and robustness of GBD estimates are dependent on quality and quantity of data used in modelling. The heterogeneity and scarcity of sub-national surveillance data from India might contribute to bias in the present study. As, the latest GBD data is available until the year 2019 and has not been updated yet, consequently, any changes in the trends of VBDs in the last three years could not be assessed.

These findings underscore the urgency of redoubling and expanding efforts to reduce and prevent the burden of VBDs in India through evidence-based interventions. Vulnerable demographic groups and geographic areas with the highest VBDs burden should be the focus of integrated control and surveillance strategies.

Our analyses highlights the national and subnational epidemiological trends of VBDs, which would help in formulating better management and surveillance programmes. The analysis from the present study must pave the way for health agencies to reinforce and expand regional and national VBD surveillance and control programmes, as well as in developing focused measures to halt the national VBD trends, identify outbreaks and manage emerging foci. It is advisable to encourage government investment in monitoring and preparedness capacities to swiftly detect and report disease outbreaks early on particularly given the potential expansion of dengue epidemic and the ongoing LF and leishmaniasis eradication efforts in India.

## Supporting information

S1 File Prevalence, incidence, DALYs and death for dengue, leishmaniasis and lymphatic filariasis for Indian states and union territories. (PDF)

## References

[1] Chala B, Hamde F. Emerging and re-emerging vector-borne infectious diseases and the challenges for control: A review. Frontiers in public health. 2021 Oct 5;9:715759. doi: 10.3389/fpubh.2021.715759 34676194 PMC8524040

[2] Wilson AL, Courtenay O, Kelly-Hope LA, Scott TW, Takken W, Torr SJ, et al. The importance of vector control for the control and elimination of vector-borne diseases. PLoS neglected tropical diseases. 2020 Jan 16;14(1):e0007831. doi: 10.1371/journal.pntd.0007831 31945061 PMC6964823

[3] Golding N, Wilson AL, Moyes CL, Cano J, Pigott DM, Velayudhan R, et al. Integrating vector control across diseases. BMC medicine. 2015 Dec;13(1):1–6. doi: 10.1186/s12916-015-0491-4 26423147 PMC4590270

[4] WHO, 2020: https://www.who.int/news-room/fact-sheets/detail/vector-borne-diseases accessed on 1st October 2022

[5] Athni TS, Shocket MS, Couper LI, Nova N, Caldwell IR, Caldwell JM, et al. The influence of vector‐borne disease on human history: socio‐ecological mechanisms. Ecology letters. 2021 Apr;24(4):829–46. doi: 10.1111/ele.13675 33501751 PMC7969392

[6] Dhiman RC, Pahwa S, Dhillon GP, Dash AP. Climate change and threat of vector-borne diseases in India: are we prepared?. Parasitology research. 2010 Mar;106:763–73. doi: 10.1007/s00436-010-1767-4 20155369

[7] Das S, Sarfraz A, Jaiswal N, Das P. Impediments of reporting dengue cases in India. Journal of infection and public health. 2017 Sep 1;10(5):494–8. doi: 10.1016/j.jiph.2017.02.004 28262571

[8] Agrawal VK, Sashindran VK. Lymphatic filariasis in India: problems, challenges and new initiatives. Medical Journal Armed Forces India. 2006 Oct 1;62(4):359–62. doi: 10.1016/S0377-1237(06)80109-7 27688542 PMC5034168

[9] Ashour A, Atia A, Akash N, Jumaa E, Alkhishrabi A. Cutaneous Leishmaniasis in Al-Jabal Al-Gharbi, Libya: Incidence and Epidemiology. Khalij-Libya Journal of Dental and Medical Research. 2022 May 27:81–5.

[10] Gupta N, Srivastava S, Jain A, Chaturvedi UC. Dengue in india. The Indian journal of medical research. 2012 Sep;136(3):373. 23041731 PMC3510884

[11] Salam N, Mustafa S, Hafiz A, Chaudhary AA, Deeba F, Parveen S. Global prevalence and distribution of coinfection of malaria, dengue and chikungunya: a systematic review. BMC public health. 2018;18(1):1–20. doi: 10.1186/s12889-018-5626-z 29879935 PMC5992662

[12] Kakkar M. Dengue fever is massively under-reported in India, hampering our response. Bmj. 2012 Dec 19;345. doi: 10.1136/bmj.e8574 23255584

[13] https://ncvbdc.mohfw.gov.in/index4.php?lang=1&level=0&linkid=431&lid=3715 (accessed on 6th June, 2023)

[14] Gubler DJ. Dengue, urbanization and globalization: the unholy trinity of the 21st century. Tropical medicine and health. 2011;39(4SUPPLEMENT):S3–11. 10.2149/tmh.2011-S05 PMC3317603 22500131

[15] Mutheneni SR, Morse AP, Caminade C, Upadhyayula SM. Dengue burden in India: recent trends and importance of climatic parameters. Emerging microbes & infections. 2017 Jan 1;6(1):1–0. doi: 10.1038/emi.2017.57 28790459 PMC5583666

[16] Palaniyandi M. and Temporal Analysis of Vector Borne Disease Epidemics for Mapping the Hotspot Region, Risk Assessment, and Control for Sustainable Health. Indian Journal of Public Health Research & Development. 2021 Oct 1;12(4).

[17] Kamgno J, Djeunga HN. Progress towards global elimination of lymphatic filariasis. The Lancet Global Health. 2020 Sep 1;8(9):e1108–9. doi: 10.1016/S2214-109X(20)30323-5 32827473

[18] Singh OP, Hsker E, Boelaert M, Sundar S. Elimination of visceral leishmaniasis on the Indian subcontinent. The Lancet infectious diseases. 2016 Dec 1;16(12):e304–9. doi: 10.1016/S1473-3099(16)30140-2 27692643 PMC5177523

[19] Dhamnetiya D, Jha RP, Shalini, Bhattacharyya K. India’s performance in controlling Visceral Leishmaniasis as compared to Brazil over past three decades: findings from global burden of disease study. Journal of Parasitic Diseases. 2021 Dec;45(4):877–86. 34789968 10.1007/s12639-021-01375-0 PMC8556458

[20] Cloots K, Marino P, Burza S, Gill N, Boelaert M, Hasker E. Visceral leishmaniasis-HIV coinfection as a predictor of increased leishmania transmission at the village level in Bihar, India. Frontiers in Cellular and Infection Microbiology. 2021 Mar 11;11:604117. doi: 10.3389/fcimb.2021.604117 33777831 PMC7993201

[21] Thakur L, Kushwaha HR, Negi A, Jain A, Jain M. Leptomonas seymouri co-infection in cutaneous leishmaniasis cases caused by Leishmania donovani from Himachal Pradesh, India. Frontiers in Cellular and Infection Microbiology. 2020 Jul 15;10:345. doi: 10.3389/fcimb.2020.00345 32760679 PMC7373763

[22] Saini P, Kumar NP, Ajithlal PM, Joji A, Rajesh KR, Reena KJ, et al. Visceral Leishmaniasis Caused by Leishmania donovani Zymodeme MON-37, Western Ghats, India. Emerging Infectious Diseases. 2020 Aug;26(8):1956. doi: 10.3201/eid2608.200557 32687040 PMC7392465

[23] Verma R, Kushwaha V, Pandey S, Thota JR, Vishwakarma P, Parmar N, et al. Leishmania donovani molecules recognized by sera of filaria infected host facilitate filarial infection. Parasitology research. 2018 Sep;117:2901–12. doi: 10.1007/s00436-018-5981-9 29946763

[24] Singh AK, Hasker E, Singh OP, Sundar S. Leishmania Donovani and Wuchereria Bancrofti co-infection in an asymptomatic population of visceral leishmaniasis. International Journal of Infectious Diseases. 2023 May 1;130:S23.

[25] Kushwaha V, Kaur S. Lymphatic filariasis and visceral leishmaniasis coinfection: A review on their epidemiology, therapeutic, and immune responses. Acta Tropica. 2021 Dec 1;224:106117. doi: 10.1016/j.actatropica.2021.106117 34464587

[26] Rana MS, Alam MM, Ikram A, Salman M, Mere MO, Usman M, et al. Emergence of measles during the COVID-19 pandemic threatens Pakistan’s children and the wider region. Nature Medicine. 2021 Jul;27(7):1127–8. doi: 10.1038/s41591-021-01430-6 34183836

[27] Kalkowska DA, Voorman A, Pallansch MA, Wassilak SG, Cochi SL, Badizadegan K, et al. The impact of disruptions caused by the COVID-19 pandemic on global polio eradication. Vaccine. 2023 Apr 6;41:A12–8. doi: 10.1016/j.vaccine.2021.04.026 33962838 PMC10045205

