# Supplementary material for: Burden of dengue, leishmaniasis and lymphatic filariasis in India and its states from 1990–2019: Analysis from the Global Burden of Disease study (GBD 2019)
Source: PLoS One. 2023 Oct 18;18(10):e0292723. doi: 10.1371/journal.pone.0292723 (PMC10584127; doi:10.1371/journal.pone.0292723)
Supplement: S1 File — (PDF) [file pone.0292723.s001.pdf]

Supporting data

Contents---Page No.

Supporting table 1 .Age and gender wise distribution of prevalence, incidence, DALYs and death for dengue, leishmaniasis and lymphatic filariasis for the year 2019------2
Supporting figure 1. Trends for prevalence, incidence, DALY and death for dengue from 1990-2019---3
Supporting figure 2. Trends for prevalence, incidence, DALY and death for leishmaniasis from 1990-2019---4
Supporting figure 3. Trends for prevalence and DALY for LF from 1990-2019---5
Supporting figure 4. Comparison between prevalence and mortality for dengue and leishmaniasis from 1990-2019--6
Supporting figure 5. Prevalence rate per 100,000 for Indian states and union territories for dengue for the years 1990 and 2019---7
Supporting figure 6. Incidence rate per 100,000 for Indian states and union territories for dengue for the years 1990 and 2019--------------------------------8
Supporting figure 7. DALY rate per 100,000 for Indian states and union territories for dengue for the years 1990 and 2019--------------------------- ---------------------9
Supporting figure 8. Death rate per 100,000 for Indian states and union territories for dengue for the years 1990 and 2019--10
Supporting figure 9. Prevalence rate per 100,000 for Indian states and union territories for leishmaniasis for the years 1990 and 2019-------------------------------------11
Supporting figure 10. Incidence rate per 100,000 for Indian states and union territories for leishmaniasis for the years 1990 and 2019-------------------12
Supporting figure 11. DALY rate per 100,000 for Indian states and union territories for leishmaniasis for the years 1990 and 2019----------------------13
Supporting figure 12. Death rate per 100,000 for Indian states and union territories for leishmaniasis for the years 1990 and 2019----------------------14
Supporting figure 13. Prevalence rate per 100,000 for Indian states and union territories for lymphatic filariasis for the years 1990 and 2019----------15
Supporting figure 14. DALY rate per 100,000 for Indian states and union territories for lymphatic filariasis for the years 1990 and 2019-----------------------------------16
Supporting table 2. Comparison of incidence data from GBD and NVBDCP (renamed NCVBDC) for dengue and leishmaniasis from 2015-2019-----------------------------17

Age	Dengue		Leishmaniasis		Lymphatic filariasis	
	Prevalence		Prevalence		Prevalence	
	Male	Female	Male	Female	Male	Female
<5	43937	44716	0	0	347237	314963
5-14	177872	178703	0	0	2936477	1949864
15-49	446184	463788	366	357	15676410	11326921
50-69	106048	121908	620	690	4860238	3737163
>70	38683	48616	332	461	1361667	1173016
Age	Incidence		Incidence		Incidence	
	Male	Female	Male	Female	Male	Female
<5	735902	735931	1295	671	0	0
5-14	2369972	2978600	2461	952	0	0
15-49	3938929	7477812	1916	756	0	0
50-69	796976	1778123	242	144	0	0
>70	168707	648208	77	42	0	0
Age	DALY		DALY		DALY	
	Male	Female	Male	Female	Male	Female
<5	73068	72391	14721	8201	0	0
5-14	87418	85887	16004	7081	170950	35842
15-49	278036	213362	21391	8791	747773	116194
50-69	82357	84730	2139	1302	210630	31051
>70	38259	51174	421	257	54375	8804
Age	Death		Death		Death	
	Male	Female	Male	Female	Male	Female
<5	756	745	170	95	0	0
5-14	726	704	200	88	0	0
15-49	3606	2484	358	149	0	0
50-69	2226	2196	67	40	0	0
>70	2427	3323	25	14	0	0

Supporting table 1. Age and gender wise distribution of prevalence, incidence, DALYs and death for dengue, leishmaniasis and lymphatic filariasis for the year 2019.

Dengue

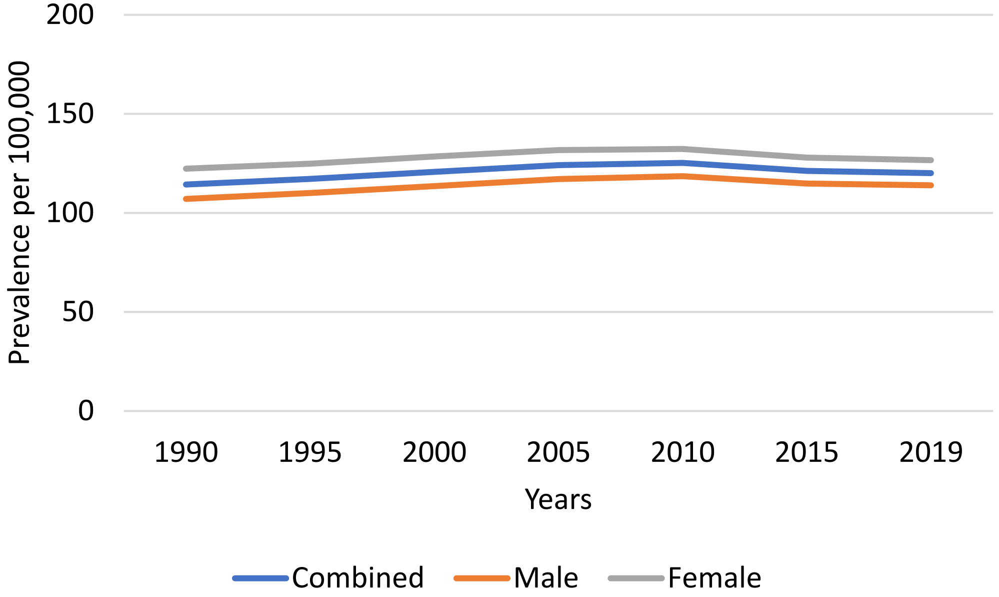
Prevalence per 100,000
200
150
100
50
0
1990
1995
2000
2005
2010
2015
2019
Years
Combined
Male
Female

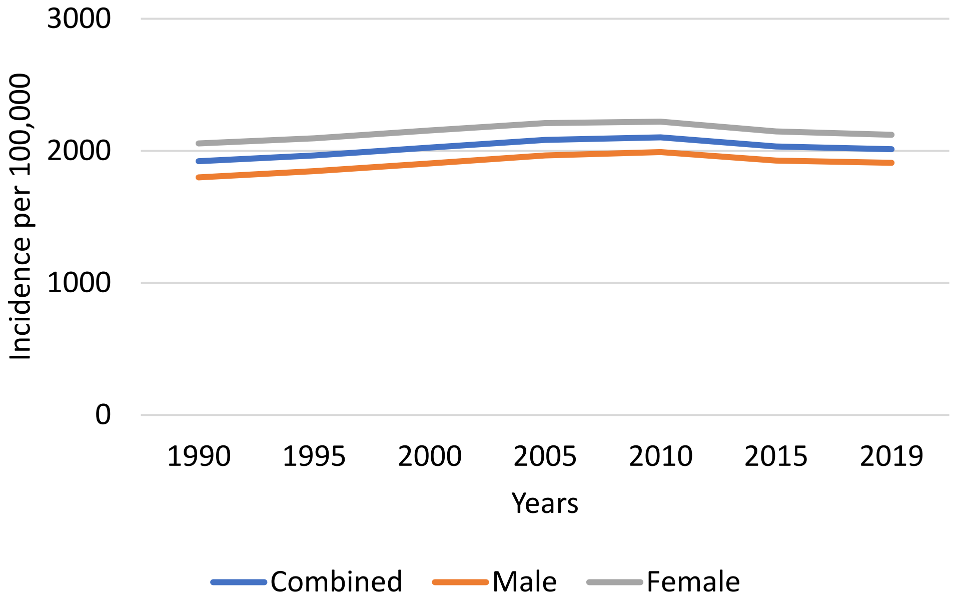
Incidence per 100,000
3000
2000
1000
0
1990
1995
2000
2005
2010
2015
2019
Years
Combined
Male
Female

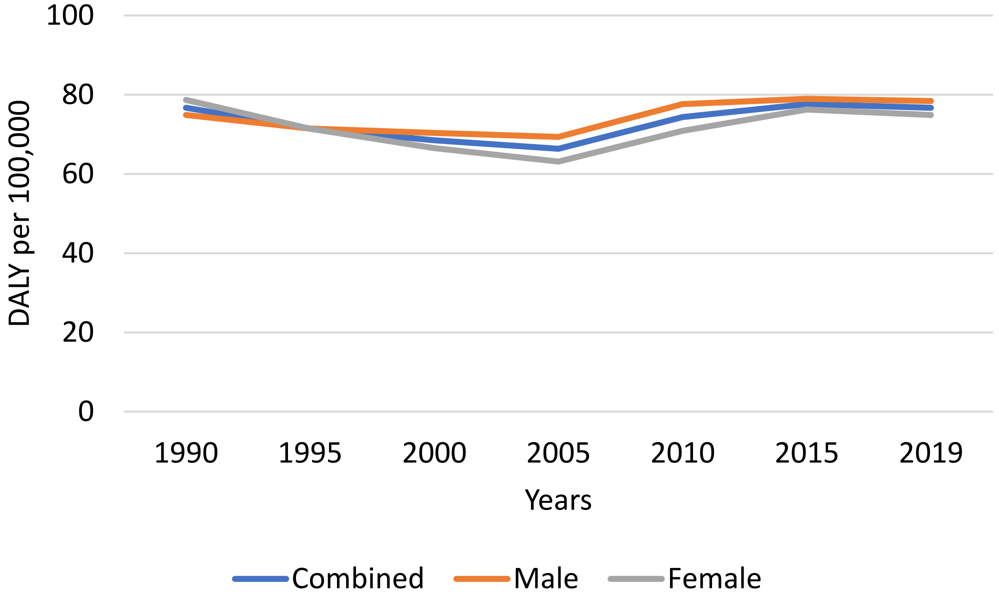
DALY per 100,000
100
80
60
40
20
0
1990
1995
2000
2005
2010
2015
2019
Years
Combined
Male
Female

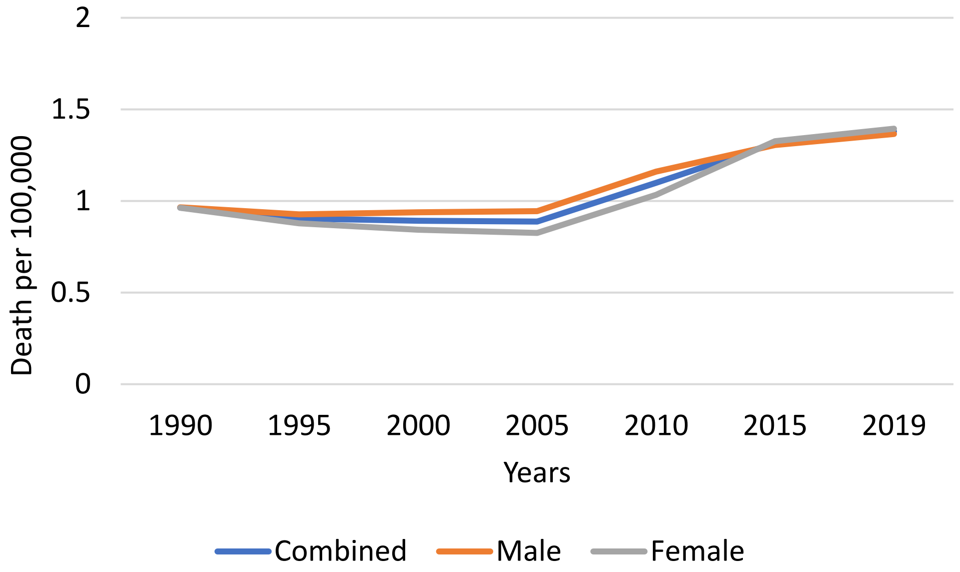
Death per 100,000
2
1.5
1
0.5
0
1990
1995
2000
2005
2010
2015
2019
Years
Combined
Male
Female

Leishmaniasis

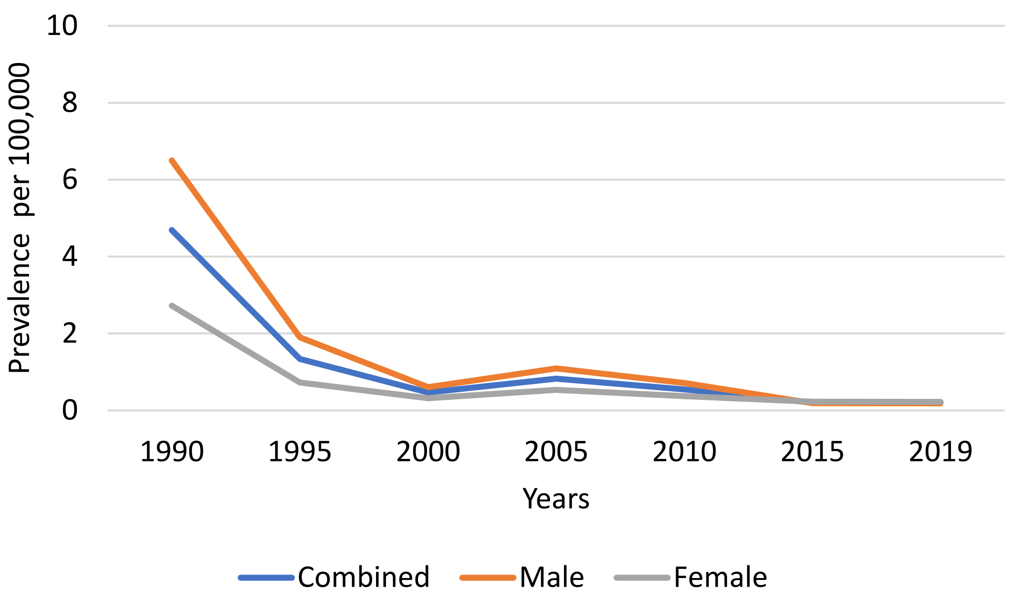

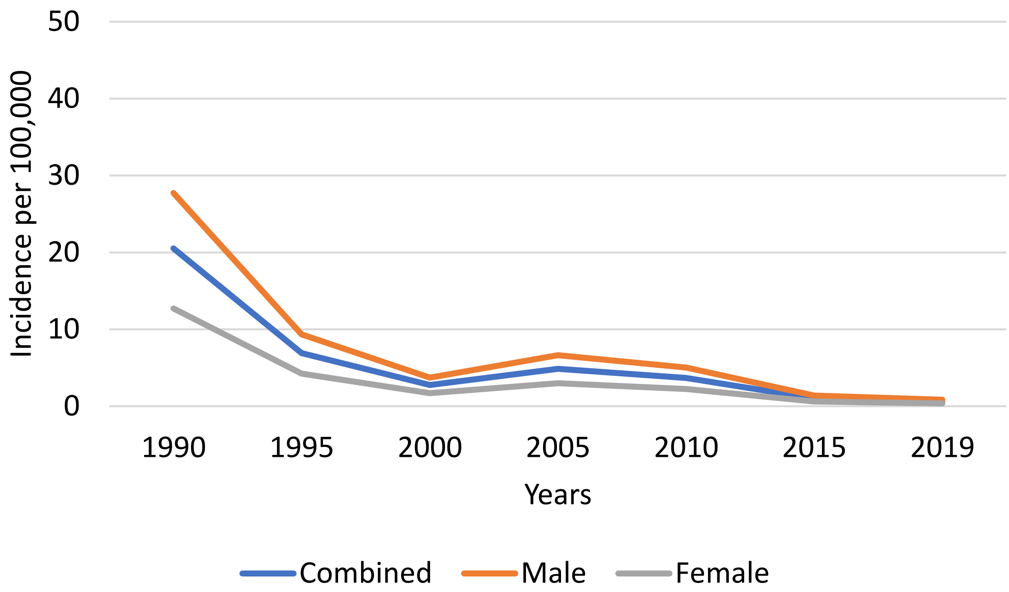

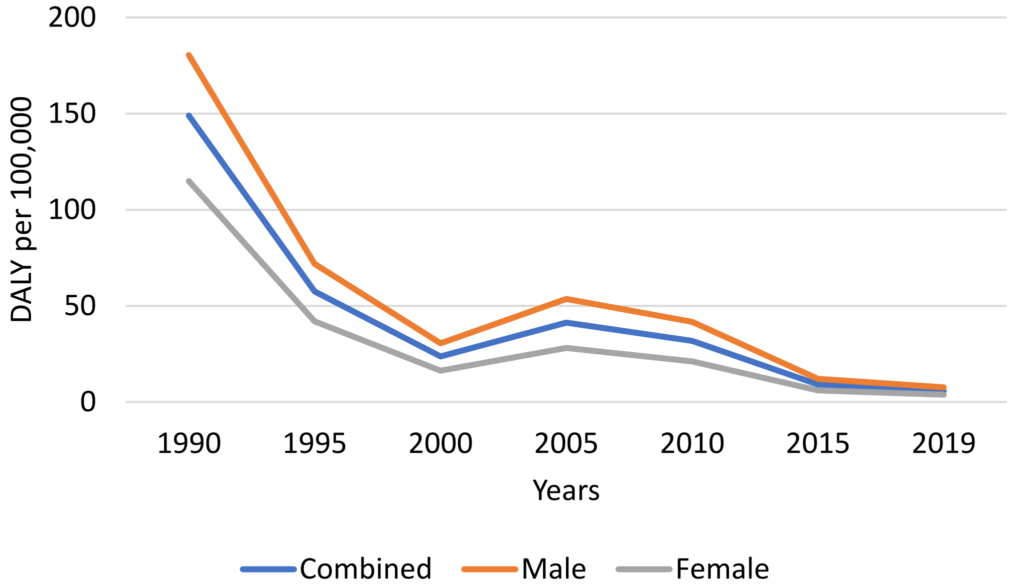

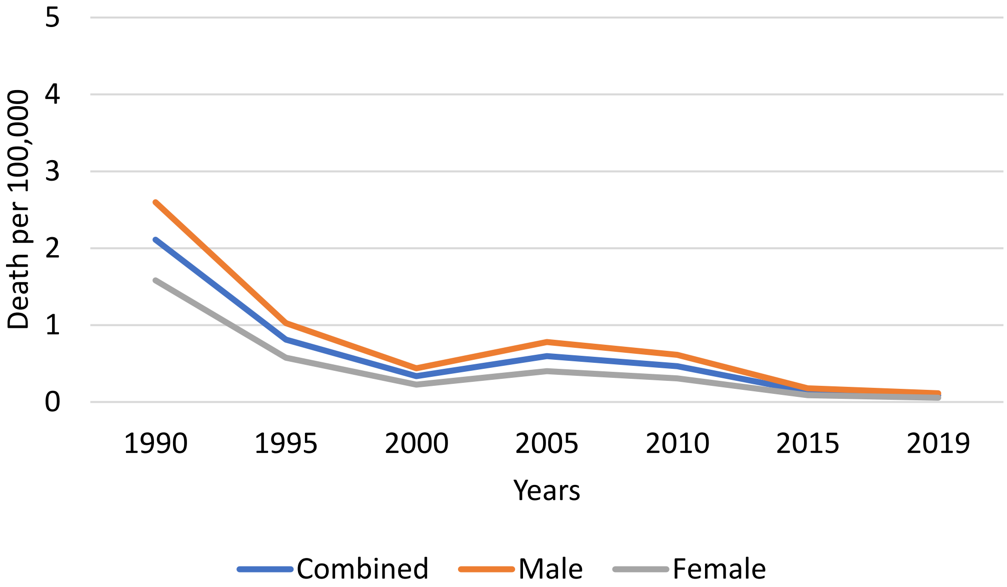

Lymphatic filariasis

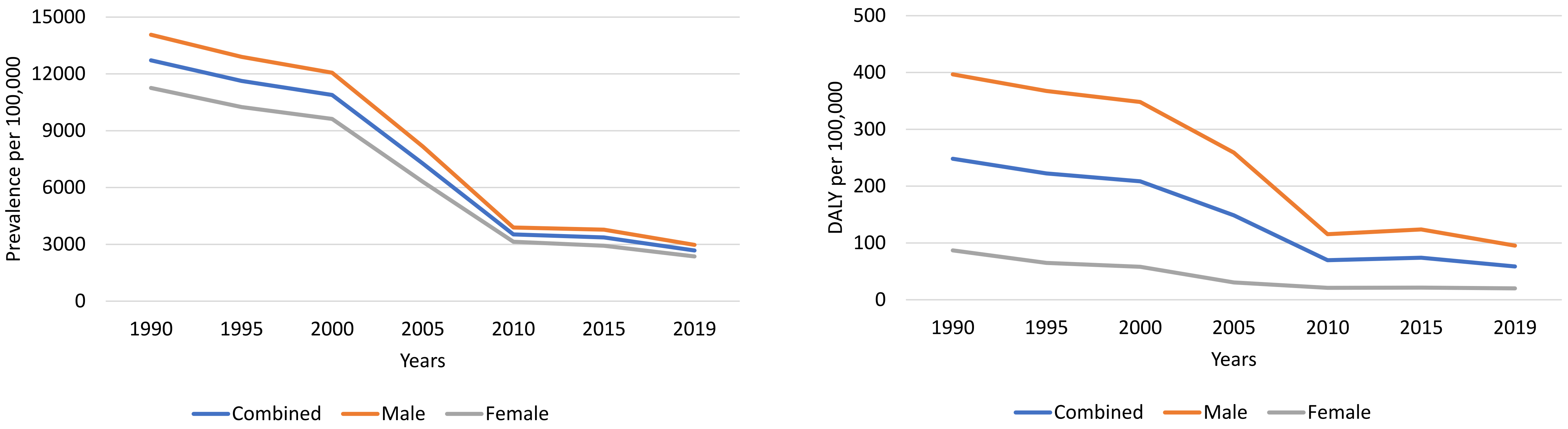

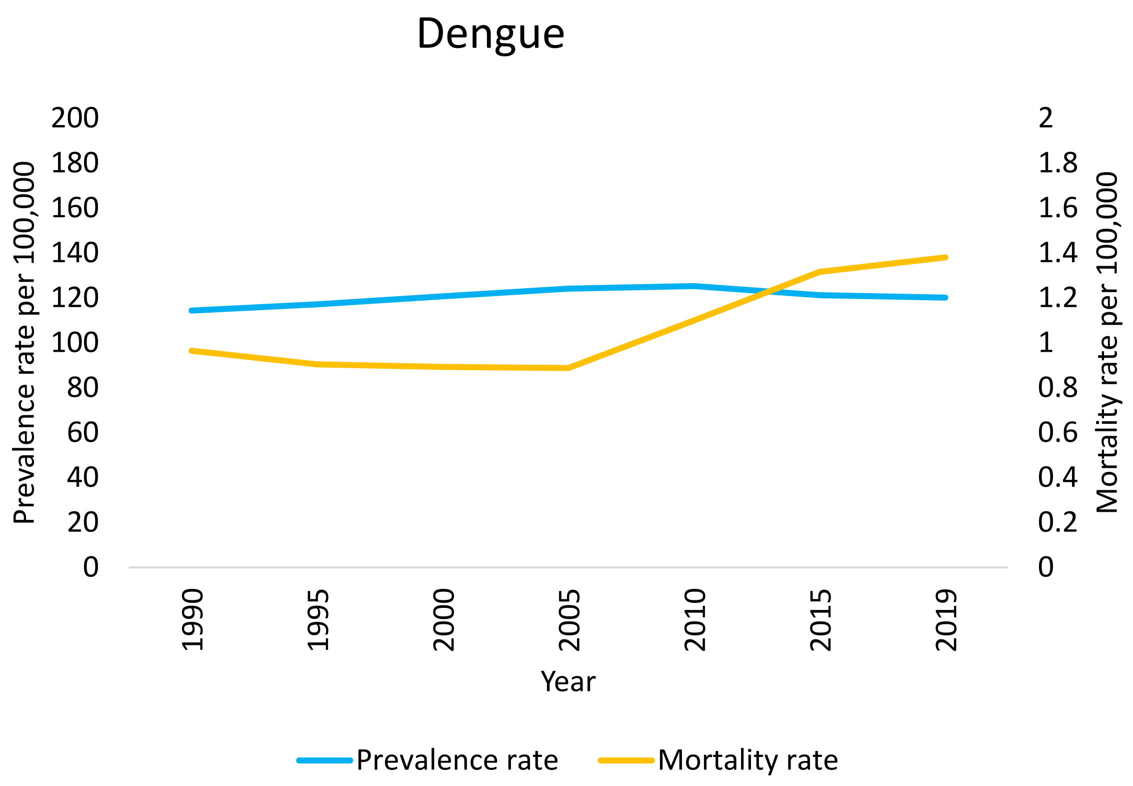
Dengue
Prevalence rate per 100,000
Mortality rate per 100,000
200
180
160
140
120
100
80
60
40
20
0
2
1.8
1.6
1.4
1.2
1
0.8
0.6
0.4
0.2
0
1990
1995
2000
2005
2010
2015
2019
Year
Prevalence rate
Mortality rate

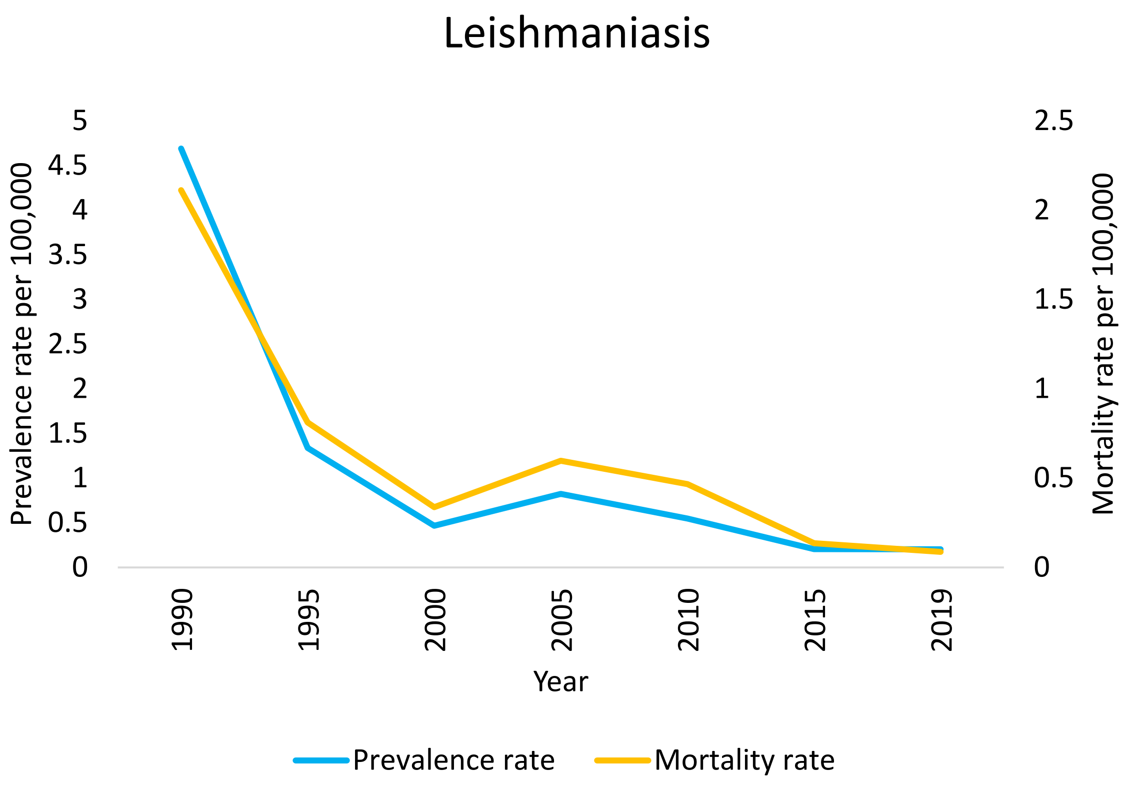
Leishmaniasis
Prevalence rate per 100,000
Mortality rate per 100,000
5
4.5
4
3.5
3
2.5
2
1.5
1
0.5
0
2.5
2
1.5
1
0.5
0
1990
1995
2000
2005
2010
2015
2019
Year
Prevalence rate
Mortality rate

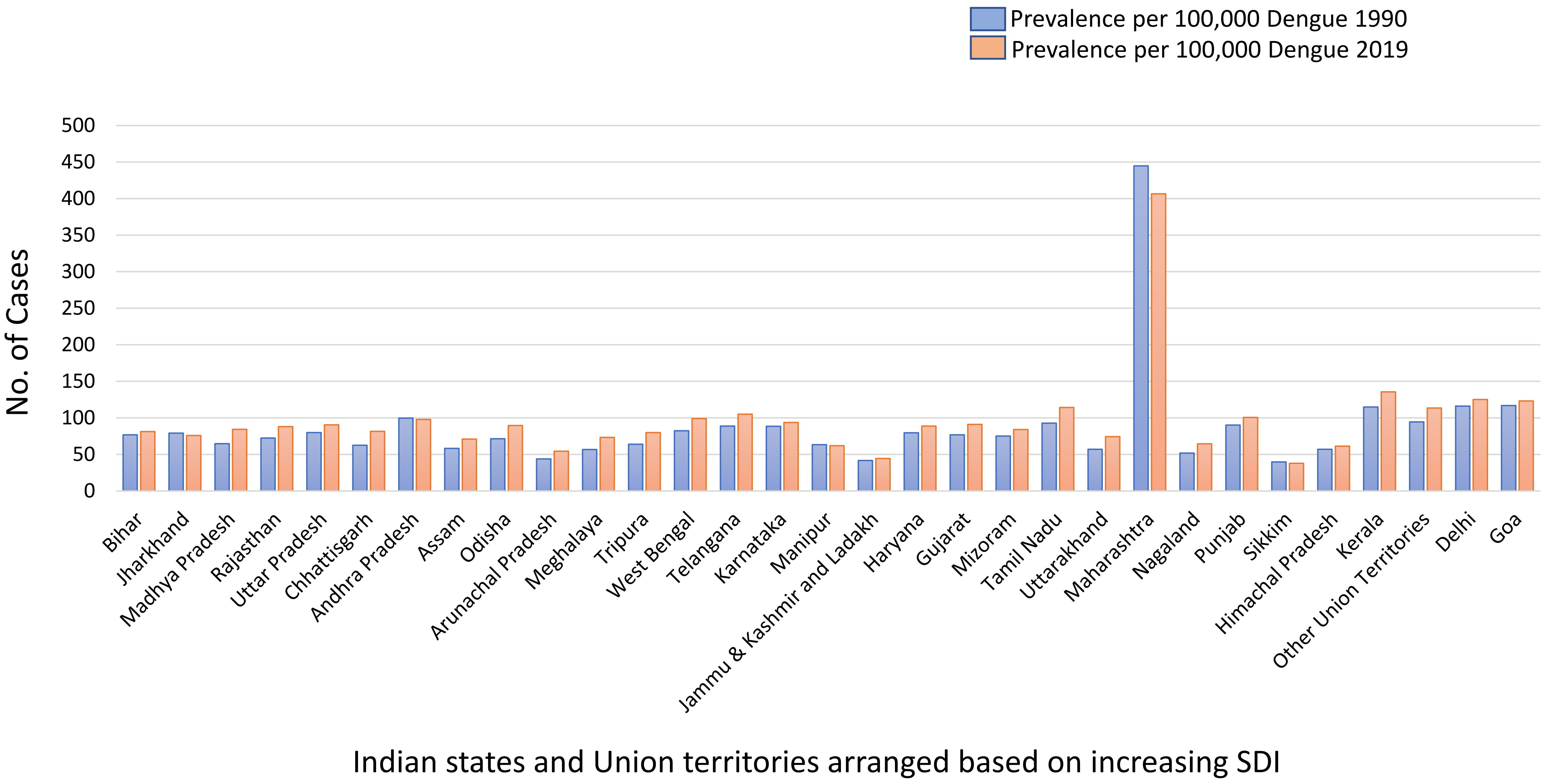

Prevalence per 100,000 Dengue 1990
Prevalence per 100,000 Dengue 2019
No. of Cases
500
450
400
350
300
250
200
150
100
50
0
Bihar
Jharkhand
Madhya Pradesh
Rajasthan
Uttar Pradesh
Chhattisgarh
Andhra Pradesh
Assam
Odisha
Arunachal Pradesh
Meghalaya
Tripura
West Bengal
Telangana
Karnataka
Manipur
Jammu & Kashmir and Ladakh
Haryana
Gujarat
Mizoram
Tamil Nadu
Uttarakhand
Maharashtra
Nagaland
Punjab
Sikkim
Himachal Pradesh
Kerala
Other Union Territories
Delhi
Goa
Indian states and Union territories arranged based on increasing SDI

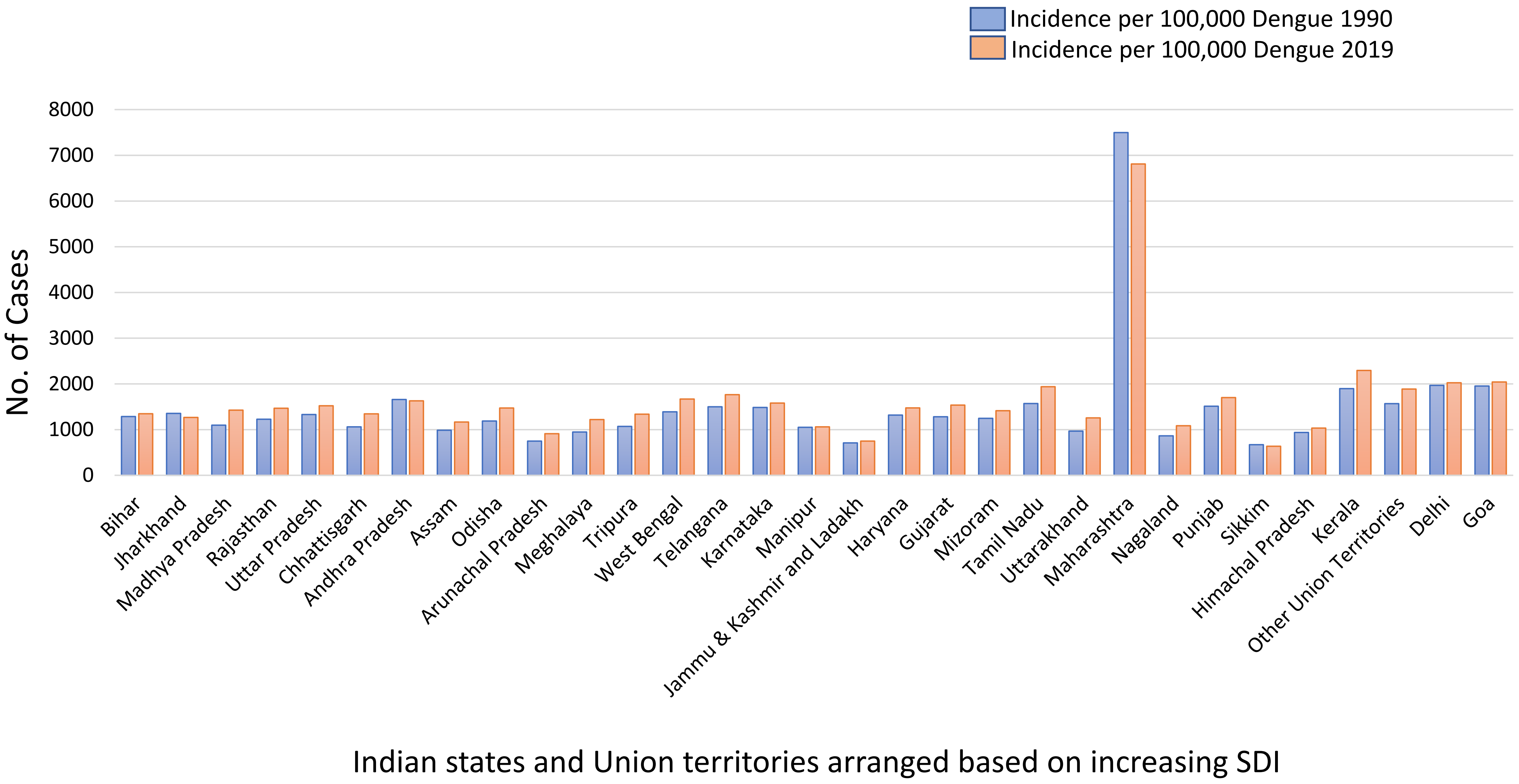

Incidence per 100,000 Dengue 1990
Incidence per 100,000 Dengue 2019
No. of Cases
8000
7000
6000
5000
4000
3000
2000
1000
0
Bihar
Jharkhand
Madhya Pradesh
Rajasthan
Uttar Pradesh
Chhattisgarh
Andhra Pradesh
Assam
Odisha
Arunachal Pradesh
Meghalaya
Tripura
West Bengal
Telangana
Karnataka
Manipur
Jammu & Kashmir and Ladakh
Haryana
Gujarat
Mizoram
Tamil Nadu
Uttarakhand
Maharashtra
Nagaland
Punjab
Sikkim
Himachal Pradesh
Kerala
Other Union Territories
Delhi
Goa
Indian states and Union territories arranged based on increasing SDI

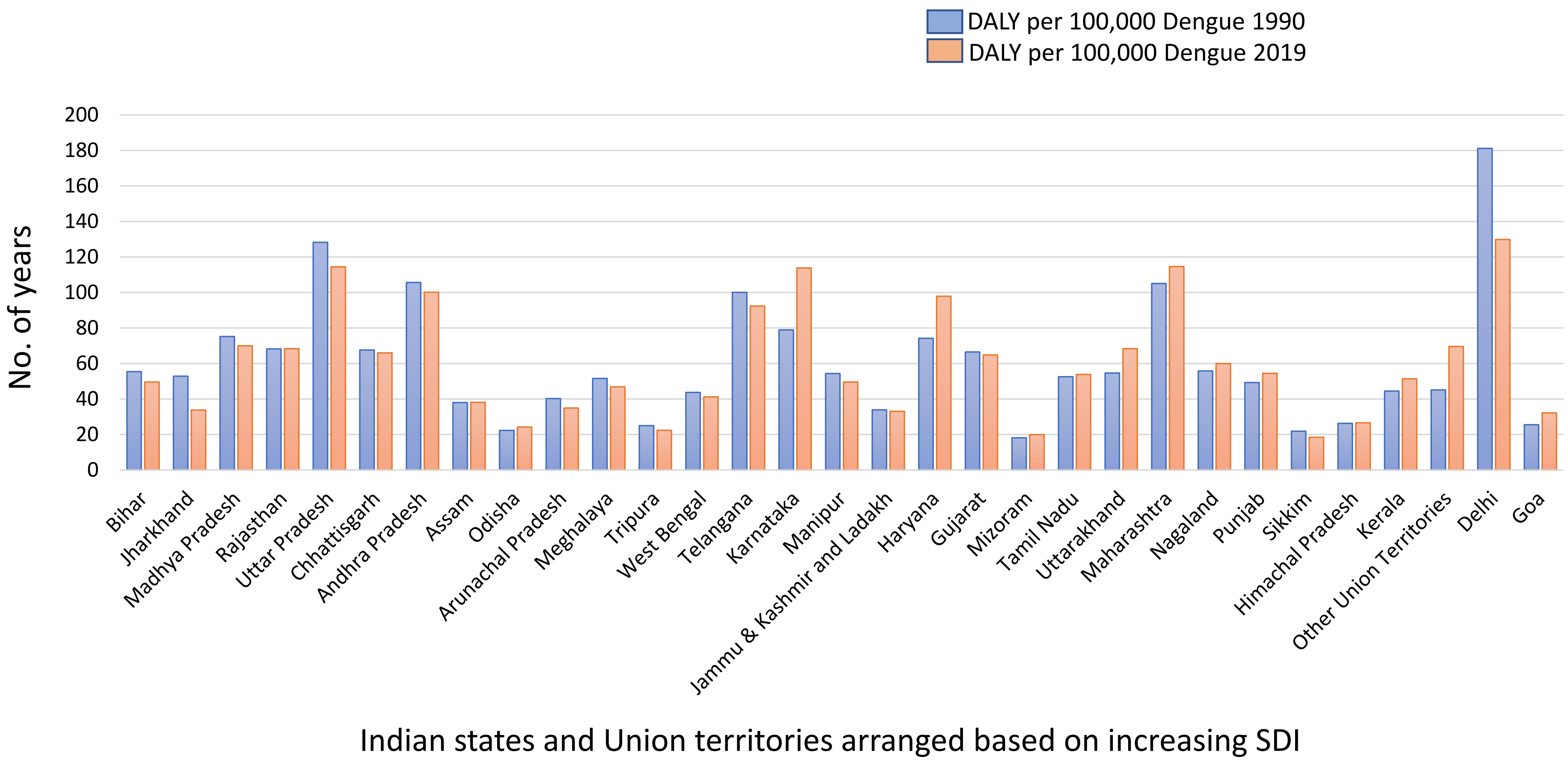

DALY per 100,000 Dengue 1990
DALY per 100,000 Dengue 2019
No. of years
200
180
160
140
120
100
80
60
40
20
0
Bihar
Jharkhand
Madhya Pradesh
Rajasthan
Uttar Pradesh
Chhattisgarh
Andhra Pradesh
Assam
Odisha
Arunachal Pradesh
Meghalaya
Tripura
West Bengal
Telangana
Karnataka
Manipur
Jammu & Kashmir and Ladakh
Haryana
Gujarat
Mizoram
Tamil Nadu
Uttarakhand
Maharashtra
Nagaland
Punjab
Sikkim
Himachal Pradesh
Kerala
Other Union Territories
Delhi
Goa
Indian states and Union territories arranged based on increasing SDI

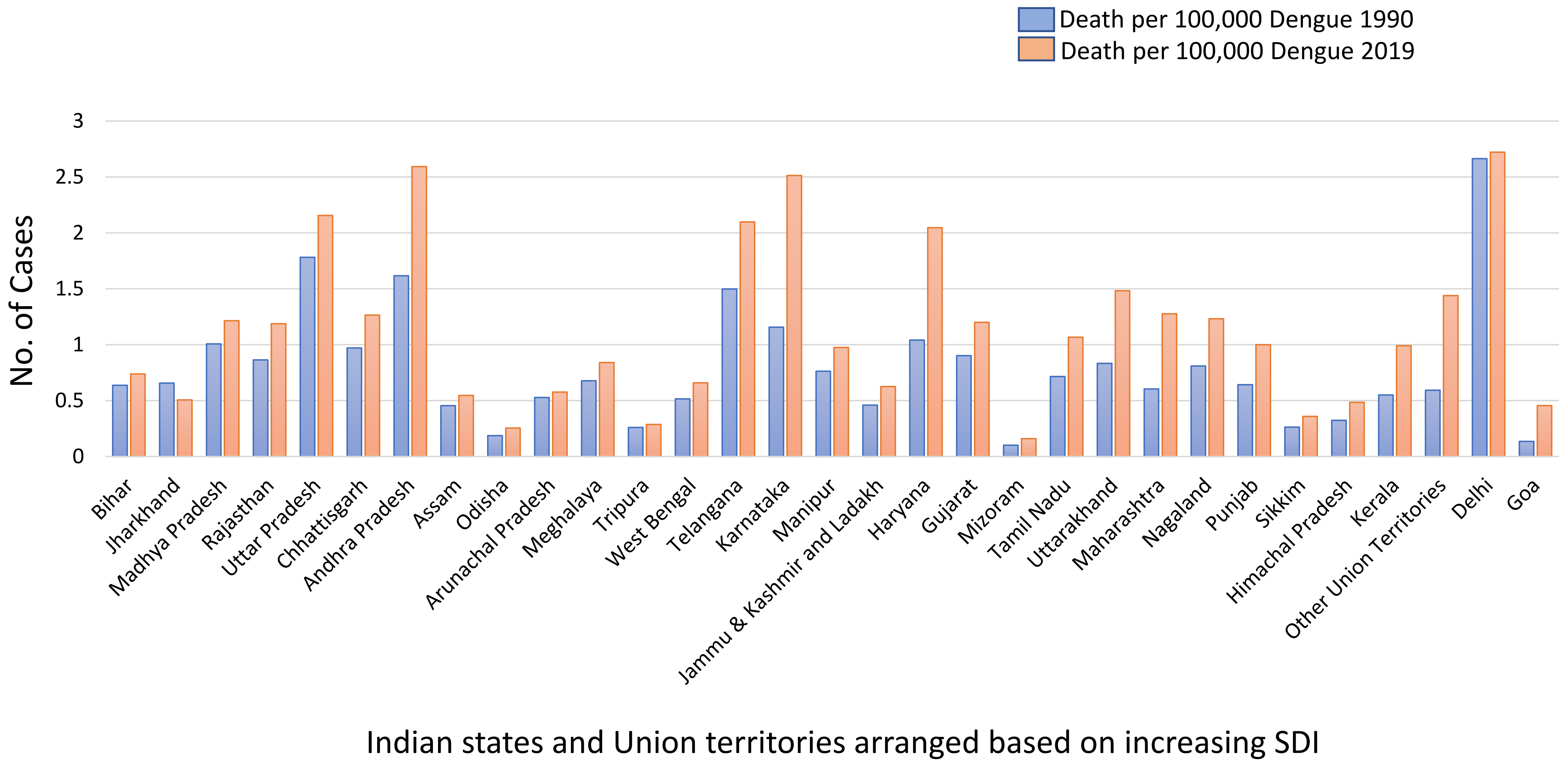
Death per 100,000 Dengue 1990
Death per 100,000 Dengue 2019
No. of Cases
3
2.5
2
1.5
1
0.5
0
Bihar
Jharkhand
Madhya Pradesh
Rajasthan
Uttar Pradesh
Chhattisgarh
Andhra Pradesh
Assam
Odisha
Arunachal Pradesh
Meghalaya
Tripura
West Bengal
Telangana
Karnataka
Manipur
Jammu & Kashmir and Ladakh
Haryana
Gujarat
Mizoram
Tamil Nadu
Uttarakhand
Maharashtra
Nagaland
Punjab
Sikkim
Himachal Pradesh
Kerala
Other Union Territories
Delhi
Goa
Indian states and Union territories arranged based on increasing SDI

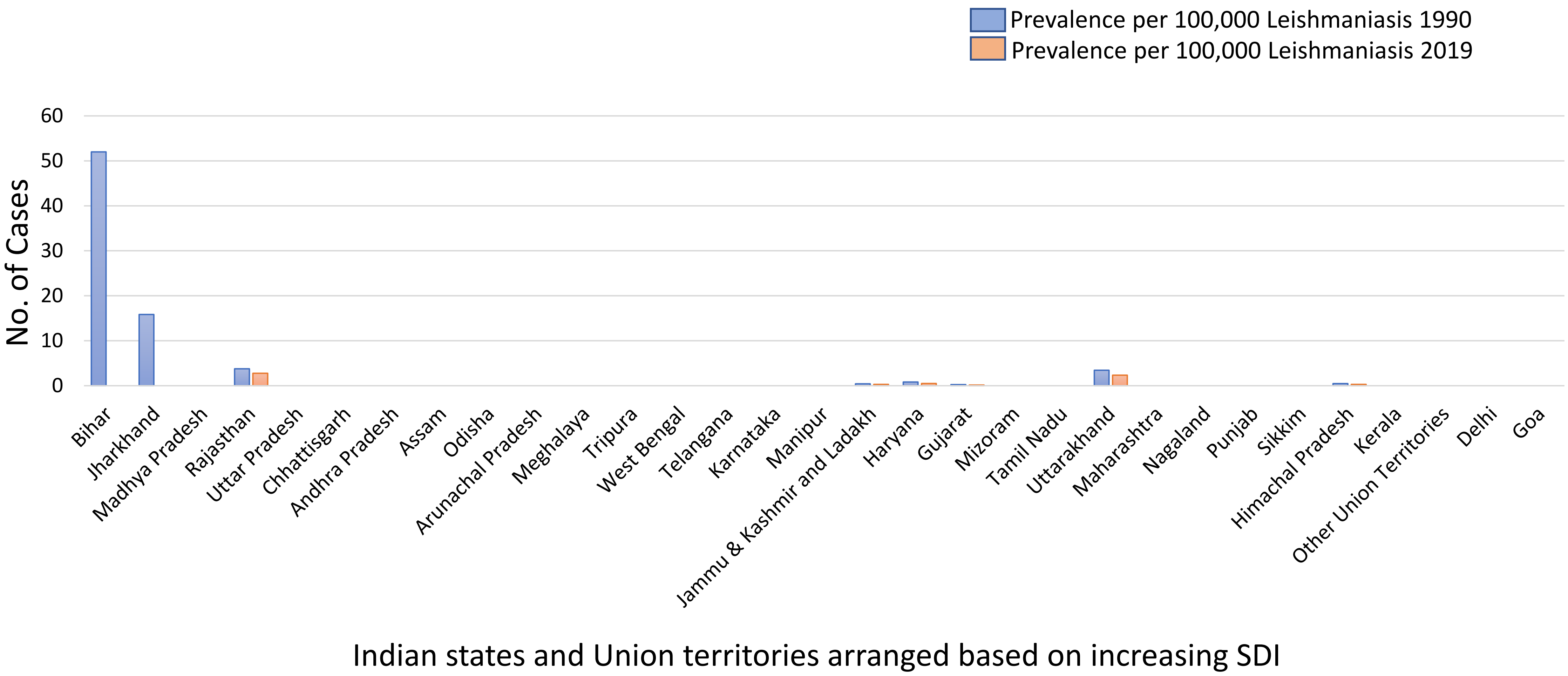
Prevalence per 100,000 Leishmaniasis 1990
Prevalence per 100,000 Leishmaniasis 2019
No. of Cases
60
50
40
30
20
10
0
Bihar
Jharkhand
Madhya Pradesh
Rajasthan
Uttar Pradesh
Chhattisgarh
Andhra Pradesh
Assam
Odisha
Arunachal Pradesh
Meghalaya
Tripura
West Bengal
Telangana
Karnataka
Manipur
Jammu & Kashmir and Ladakh
Haryana
Gujarat
Mizoram
Tamil Nadu
Uttarakhand
Maharashtra
Nagaland
Punjab
Sikkim
Himachal Pradesh
Kerala
Other Union Territories
Delhi
Goa
Indian states and Union territories arranged based on increasing SDI

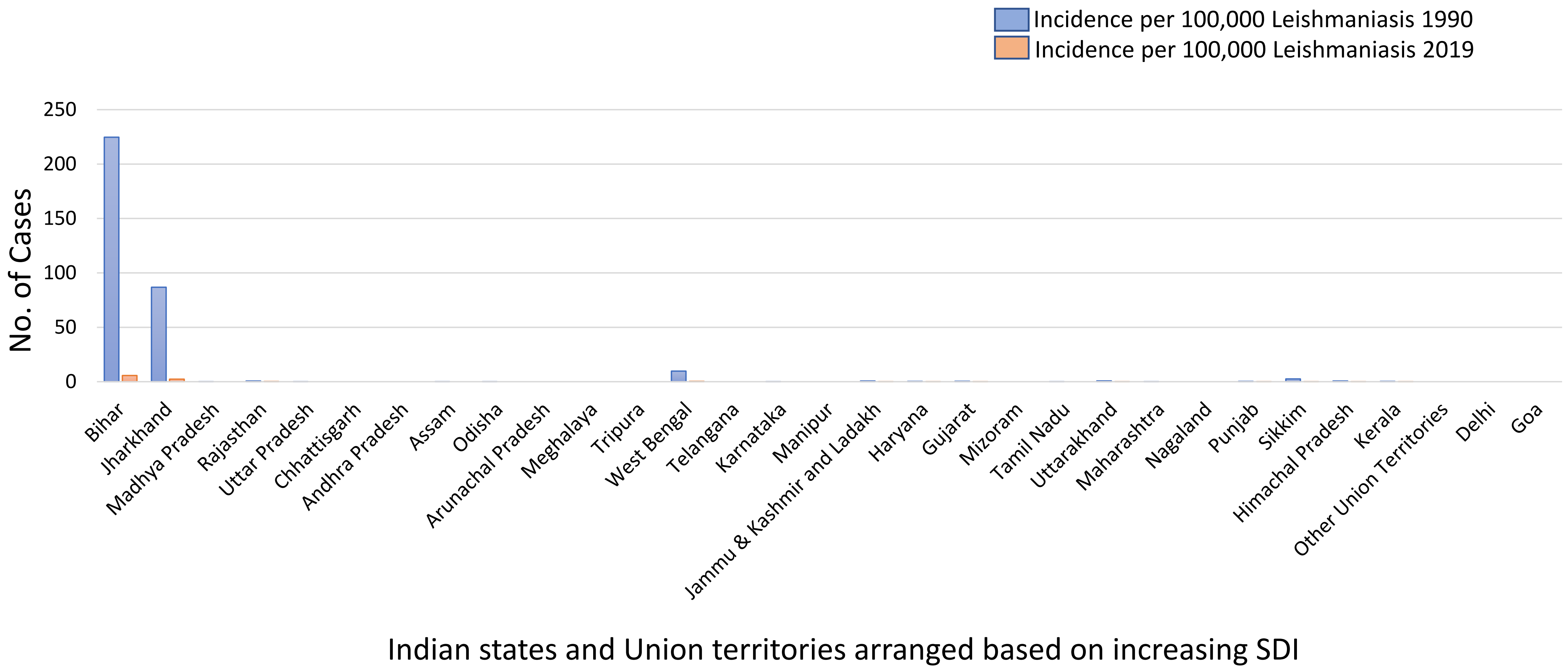
Incidence per 100,000 Leishmaniasis 1990
Incidence per 100,000 Leishmaniasis 2019
No. of Cases
250
200
150
100
50
0
Bihar
Jharkhand
Madhya Pradesh
Rajasthan
Uttar Pradesh
Chhattisgarh
Andhra Pradesh
Assam
Odisha
Arunachal Pradesh
Meghalaya
Tripura
West Bengal
Telangana
Karnataka
Manipur
Jammu & Kashmir and Ladakh
Haryana
Gujarat
Mizoram
Tamil Nadu
Uttarakhand
Maharashtra
Nagaland
Punjab
Sikkim
Himachal Pradesh
Kerala
Other Union Territories
Delhi
Goa
Indian states and Union territories arranged based on increasing SDI

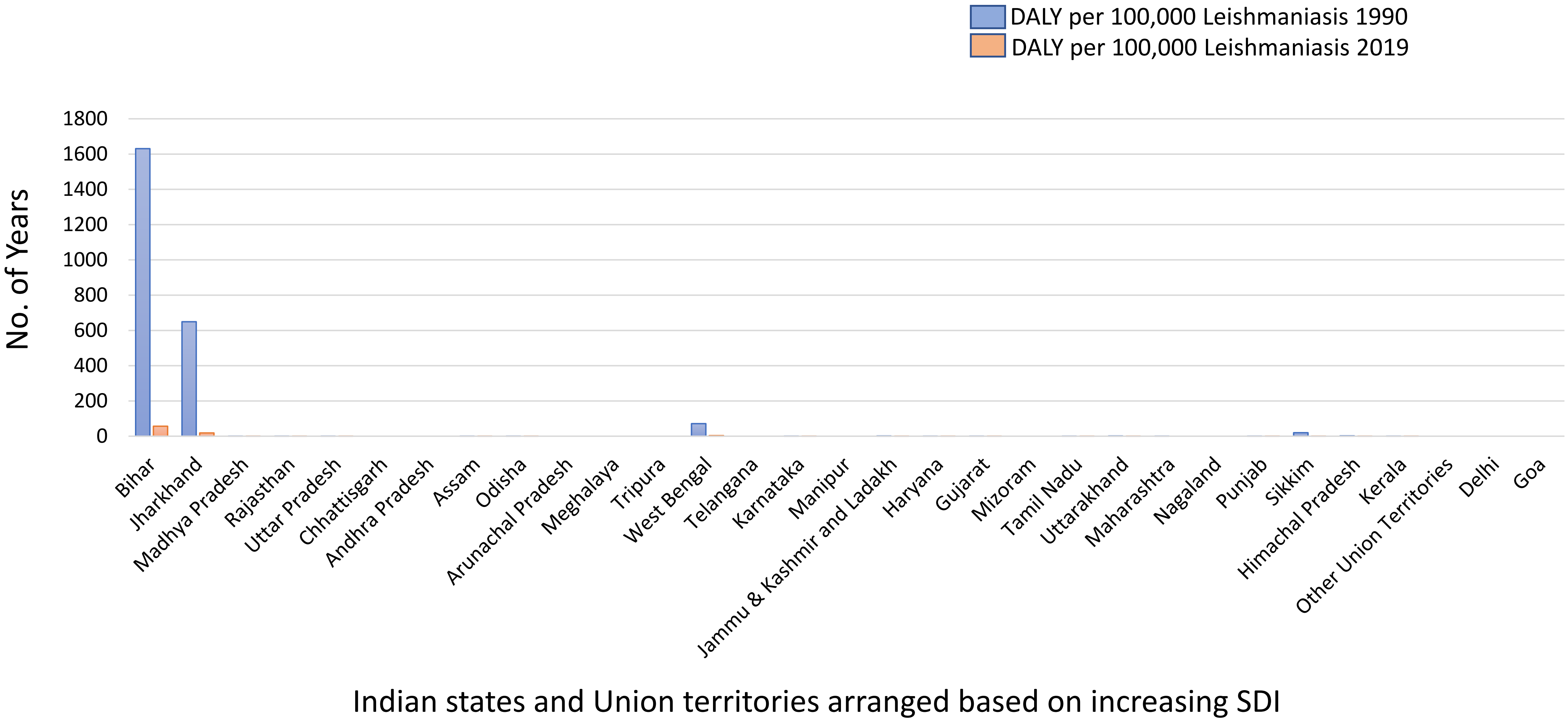
DALY per 100,000 Leishmaniasis 1990
DALY per 100,000 Leishmaniasis 2019
No. of Years
1800
1600
1400
1200
1000
800
600
400
200
0
Bihar
Jharkhand
Madhya Pradesh
Rajasthan
Uttar Pradesh
Chhattisgarh
Andhra Pradesh
Assam
Odisha
Arunachal Pradesh
Meghalaya
Tripura
West Bengal
Telangana
Karnataka
Manipur
Jammu & Kashmir and Ladakh
Haryana
Gujarat
Mizoram
Tamil Nadu
Uttarakhand
Maharashtra
Nagaland
Punjab
Sikkim
Himachal Pradesh
Kerala
Other Union Territories
Delhi
Goa
Indian states and Union territories arranged based on increasing SDI

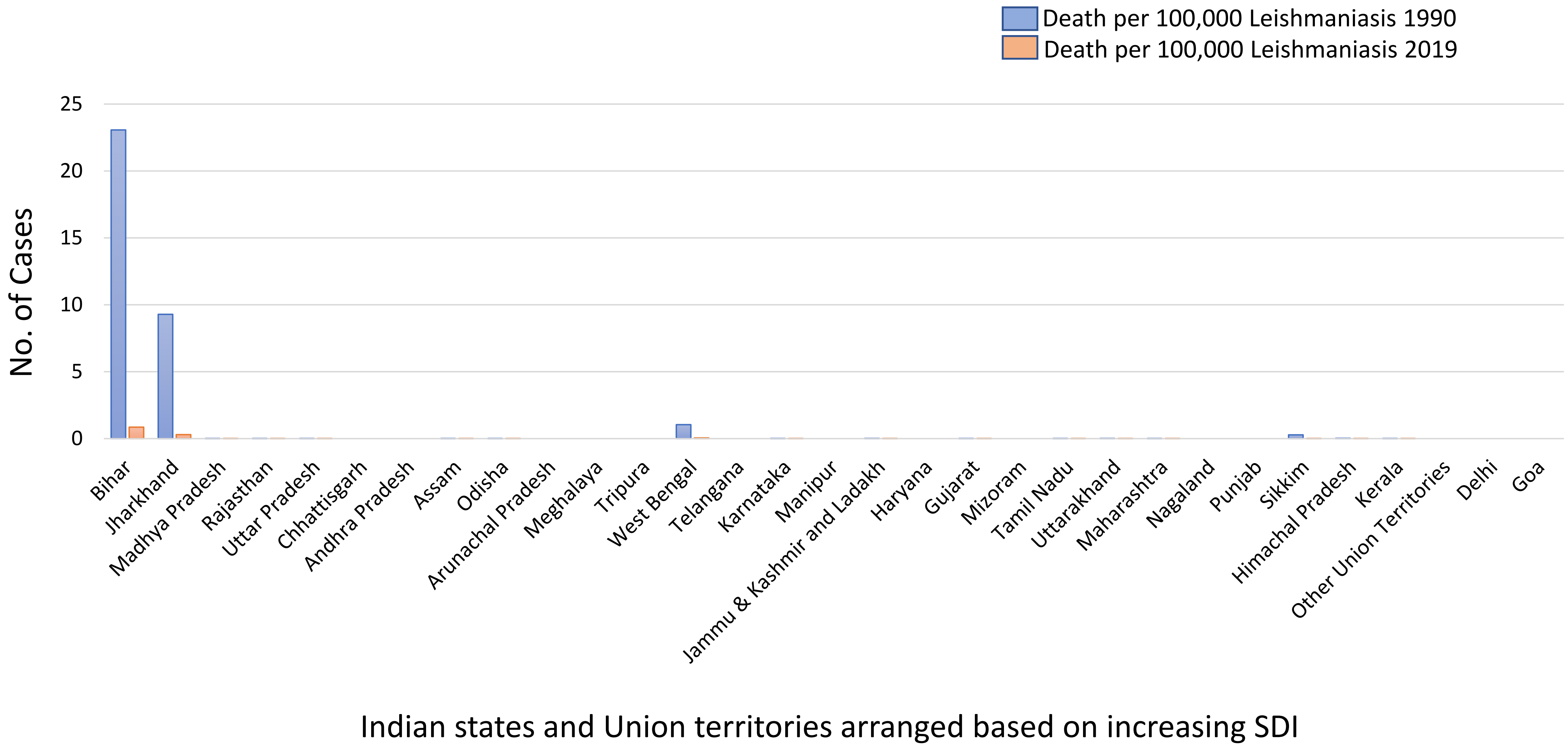
Death per 100,000 Leishmaniasis 1990
Death per 100,000 Leishmaniasis 2019
No. of Cases
25
20
15
10
5
0
Bihar
Jharkhand
Madhya Pradesh
Rajasthan
Uttar Pradesh
Chhattisgarh
Andhra Pradesh
Assam
Odisha
Arunachal Pradesh
Meghalaya
Tripura
West Bengal
Telangana
Karnataka
Manipur
Jammu & Kashmir and Ladakh
Haryana
Gujarat
Mizoram
Tamil Nadu
Uttarakhand
Maharashtra
Nagaland
Punjab
Sikkim
Himachal Pradesh
Kerala
Other Union Territories
Delhi
Goa
Indian states and Union territories arranged based on increasing SDI

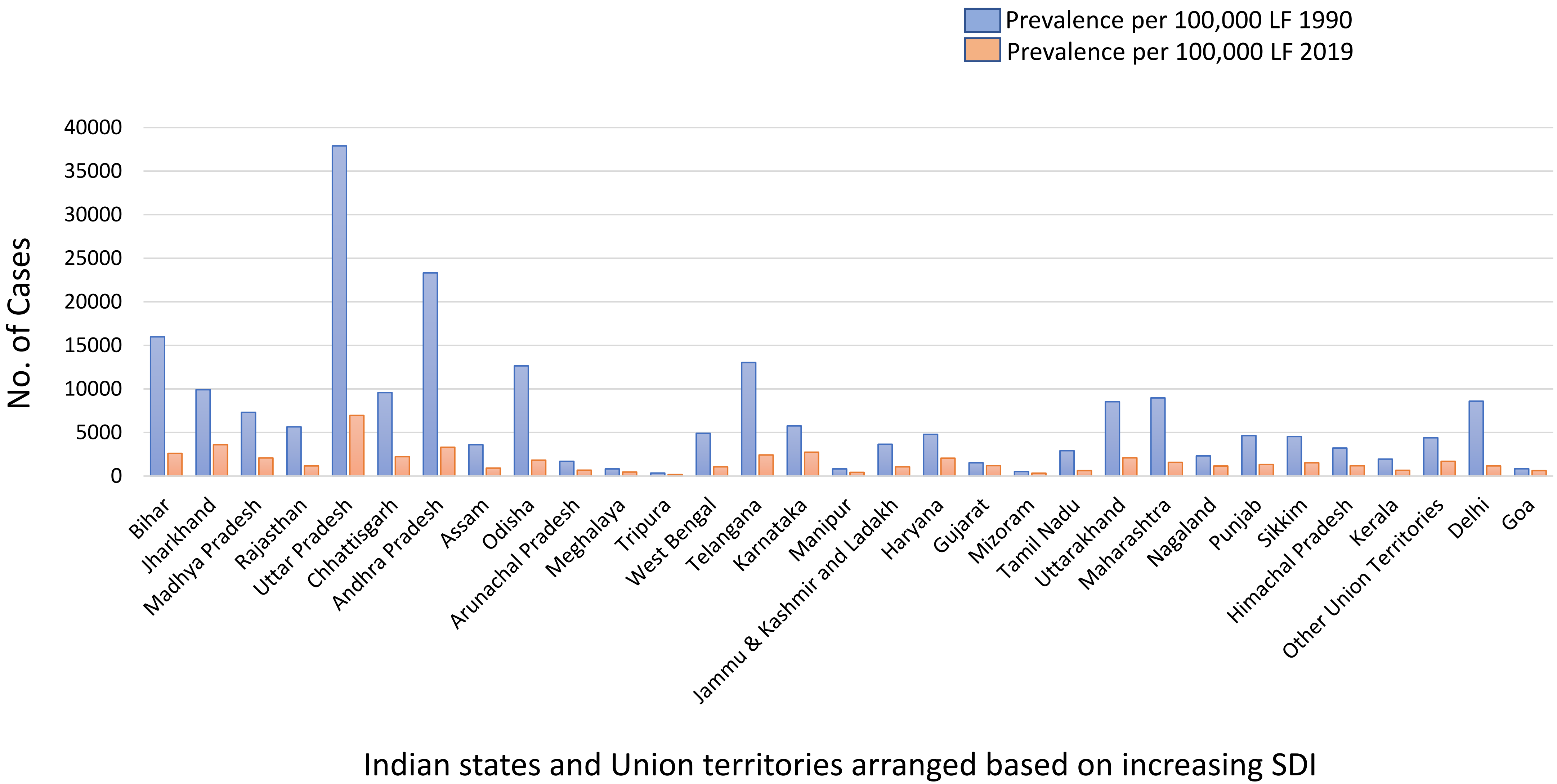

Prevalence per 100,000 LF 1990
Prevalence per 100,000 LF 2019
No. of Cases
40000
35000
30000
25000
20000
15000
10000
5000
0
Bihar
Jharkhand
Madhya Pradesh
Rajasthan
Uttar Pradesh
Chhattisgarh
Andhra Pradesh
Assam
Odisha
Arunachal Pradesh
Meghalaya
Tripura
West Bengal
Telangana
Karnataka
Manipur
Jammu & Kashmir and Ladakh
Haryana
Gujarat
Mizoram
Tamil Nadu
Uttarakhand
Maharashtra
Nagaland
Punjab
Sikkim
Himachal Pradesh
Kerala
Other Union Territories
Delhi
Goa
Indian states and Union territories arranged based on increasing SDI

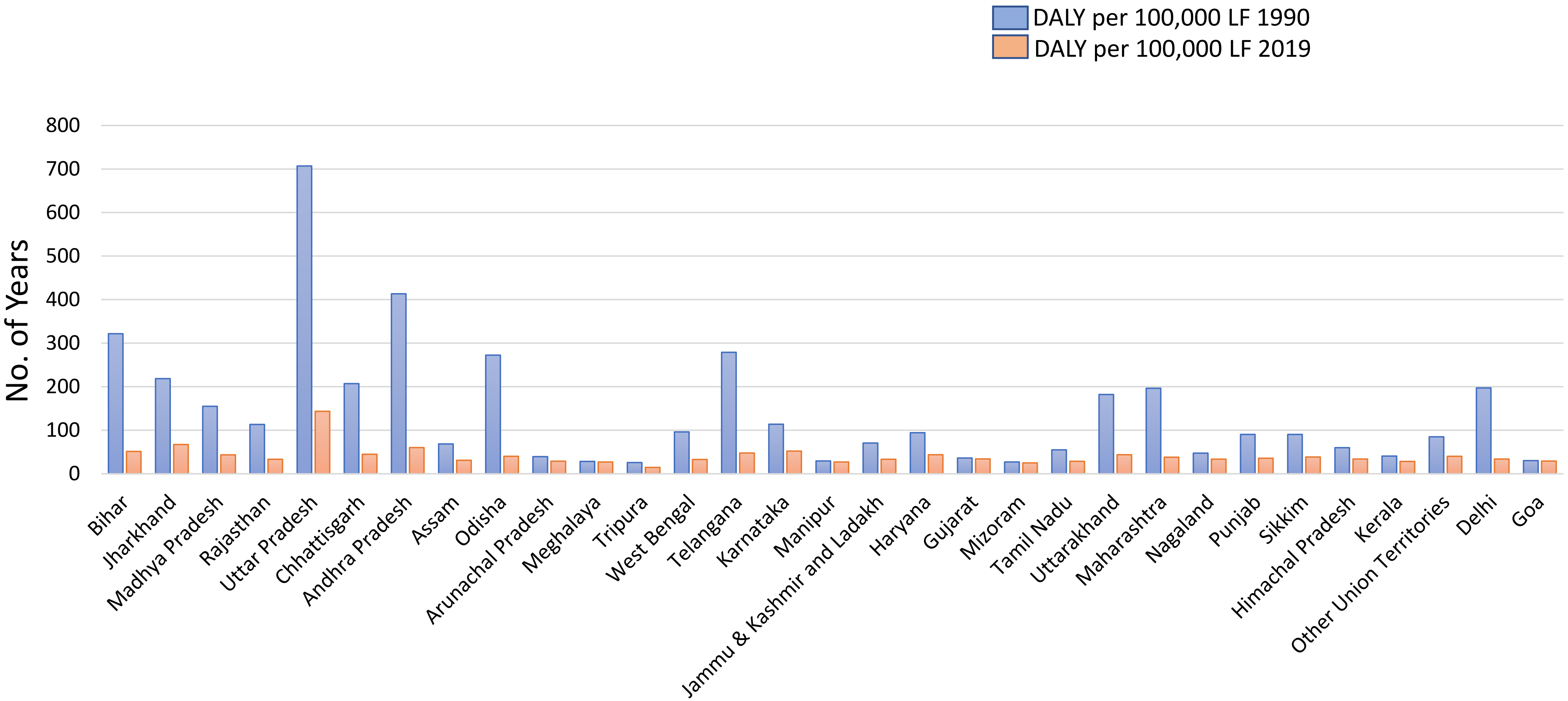

Indian states and Union territories arranged based on increasing SDI

Dengue

	2019		2018		2017		2016		2015	
**States**	GBD	NVBDCP	GBD	NVBDCP	GBD	NVBDCP	GBD	NVBDCP	GBD	NVBDCP
Jammu & Kashmir and Ladakh	104833	439	100226	214	97990.5	488	98654.4	79	99426.4	153
Arunachal Pradesh	15655.8	123	13924.4	1	13056.1	18	13126.7	3417	13268.2	1933
Uttarakhand	148560	10622	148230	689	146357	849	141125	2146	135952	1655
Himachal Pradesh	78625.3	344	75742.7	4672	73226.1	452	70137.7	322	68073.2	19
Other Union Territories	71610	4600	68334.9	1598	66236.9	7834	66917.6	6078	68027.6	3209
Andhra Pradesh	882679	5286	1021900	4011	1083028	4925	1069388	3417	1045128	3159
Assam	420471	196	441322	166	441949	5024	412526	6157	384716	1076
Delhi	393774	5077	402407	7136	407253	9271	420496	4431	431712	15867
Goa	31221.5	992	34091.6	335	35641.3	235	35844.8	150	35688.7	293
Haryana	429513	1207	422401	1898	416570	4550	411691	2493	408782	9921
Karnataka	1074655	16986	1118759	4427	1153943	17844	1224709	6083	1278571	5077
Kerala	802546	4652	735135	4083	708081	19994	717670	7439	733419	4075
Maharashtra	8493905	14907	8402179	11011	8313394	7829	8142779	6792	7980214	4936
Manipur	37190.3	359	35897	14	34860.4	193	34347.2	51	34045.4	52
Meghalaya	41710	82	37556.6	44	35680.2	52	36909.3	172	38234	13
Mizoram	18026.2	42	17603.2	68	17219.1	136	16758.2	580	16420.4	43
Nagaland	21191.8	8	21579.2	369	21772.1	357	22286.5	142	22782.2	21
Odisha	686819	3758	703618	5198	696283	4158	640180	8380	588511	2450
Punjab	528569	10289	597329	14980	620389	15298	585257	10439	548109	14128
Rajasthan	1178958	13706	1114554	9587	1070194	8427	1041259	5292	1021256	4043
Sikkim	4196.24	444	4471.14	320	4576.85	312	4543.39	82	4492.81	21
Tamil Nadu	1548184	8527	1407547	4486	1350212	23294	1413659	2531	1486165	4535
Tripura	53869.1	114	54399.6	100	54175.5	127	53467.2	0	52615.8	40
West Bengal	1662289	NR	1779677	NR	1802155	37746	1678377	22865	1554729	8516
Bihar	1645021	6712	1640171	2142	1630452	1854	1625060	1912	1614281	1771
Chhattisgarh	426355	722	380787	2674	353625	444	340519	356	331629	384
Jharkhand	480019	825	480847	463	480301	710	489182	414	496796	102
Madhya Pradesh	1264831	4189	1198646	4506	1153840	2666	1118522	3150	1092080	2108
Uttar Pradesh	3696425	10557	3637412	3829	3600116	3092	3627009	15033	3640151	2892
Gujarat	1063206	18219	1132639	7579	1147486	4753	1085778	8028	1022085	5590
Telangana	687061	13331	667233	4592	660274	5369	676691	4037	690185	1831

Leishmaniasis

States	2019		2018		2017		2016		2015	
	GBD	NVBDCP	GBD	NVBDCP	GBD	NVBDCP	GBD	NVBDCP	GBD	NVBDCP
Jammu & Kashmir and Ladakh	21.5	0	21.3	0	21.3	0	22.7	0	24.8	0
Uttarakhand	15.2	0	15.1	0	15.0	2	16.1	2	17.9	3
Himachal Pradesh	9.7	0	9.7	0	9.7	0	10.2	0	11.1	0
Assam	0.5	0	0.5	0	0.6	0	0.7	0	0.8	1
Haryana	35.4	0	35.1	0	34.9	0	37.2	0	40.5	0
Karnataka	0.5	0	0.5	0	0.6	0	0.7	0	0.8	0
Kerala	39.2	4	39.2	6	39.3	0	42.1	2	46.1	4
Maharashtra	0.5	0	0.5	0	0.6	0	0.7	0	0.8	0
Odisha	0.5	0	0.5	0	0.6	0	0.6	0	0.8	0
Punjab	38.1	0	37.9	0	37.7	0	39.9	0	43.1	1
Rajasthan	154.7	0	153.2	0	151.7	0	162.7	0	179.2	0
Sikkim	0.5	0	0.5	0	0.6	0	0.7	1	0.8	5
Tamil Nadu	0.5	0	0.5	0	0.6	0	0.6	0	0.8	0
West Bengal	309.8	87	325.8	95	357.3	156	410.5	179	496.3	576
Bihar	6914.3	2416	7262.2	3423	7978.9	4127	9169.9	4773	11089.2	6517
Jharkhand	908.6	541	954.5	752	1048.7	1358	1205.5	1185	1458.2	1262
Madhya Pradesh	0.5	0	0.5	0	0.6	0	0.6	0	0.8	0
Uttar Pradesh	6.4	97	6.7	110	7.4	115	8.5	107	10.2	131
Gujarat	98.4	0	97.4	0	96.4	0	102.1	0	110.5	0

Supporting table 2. Comparison of incidence data from GBD and NVBDCP (renamed NCVBDC) for dengue and leishmaniasis from 2015-2019.
